# Network analysis of α-synuclein pathology progression reveals p21-activated kinases as regulators of vulnerability

**DOI:** 10.1101/2024.10.22.619411

**Published:** 2024-10-22

**Authors:** Naman Vatsa, Julia K. Brynildsen, Thomas M. Goralski, Kevin Kurgat, Lindsay Meyerdirk, Libby Breton, Daniella DeWeerd, Laura Brasseur, Lisa Turner, Katelyn Becker, Kristin L. Gallik, Dani S. Bassett, Michael X. Henderson

**Affiliations:** 1 Department of Neurodegenerative Science, Van Andel Institute, Grand Rapids, MI, USA; 2 Aligning Science Across Parkinson’s (ASAP) Collaborative Research Network, Chevy Chase, MD, USA; 3 Department of Bioengineering, University of Pennsylvania, Philadelphia, PA, USA; 4 Department of Electrical & Systems Engineering, University of Pennsylvania, Philadelphia, PA, USA; 5 Department of Physics & Astronomy, University of Pennsylvania, Philadelphia, PA, USA; 6 Department of Neurology, University of Pennsylvania, Philadelphia, PA, USA; 7 Department of Psychiatry, University of Pennsylvania, Philadelphia, PA, USA; 8 Santa Fe Institute, Santa Fe, NM, USA; 9 Montreal Neurological Institute, McGill University, Montreal, Quebec, Canada; 10 Van Andel Institute, Grand Rapids, MI, USA; 11 Lead Contact

**Keywords:** Network vulnerability, p21-activated kinase, PAK5, PAK6, Snca, mitochondria, computational modeling

## Abstract

α-Synuclein misfolding and progressive accumulation drives a pathogenic process in Parkinson’s disease. To understand cellular and network vulnerability to α-synuclein pathology, we developed a framework to quantify network-level vulnerability and identify new therapeutic targets at the cellular level. Full brain α-synuclein pathology was mapped in mice over 9 months. Empirical pathology data was compared to theoretical pathology estimates from a diffusion model of pathology progression along anatomical connections. Unexplained variance in the model enabled us to derive regional vulnerability that we compared to regional gene expression. We identified gene expression patterns that relate to regional vulnerability, including 12 kinases that were enriched in vulnerable regions. Among these, an inhibitor of group II PAKs demonstrated protection from neuron death and α-synuclein pathology, even after delayed compound treatment. This study provides a framework for the derivation of cellular vulnerability from network-based studies and identifies a promising therapeutic pathway for Parkinson’s disease.

## Graphical Abstract

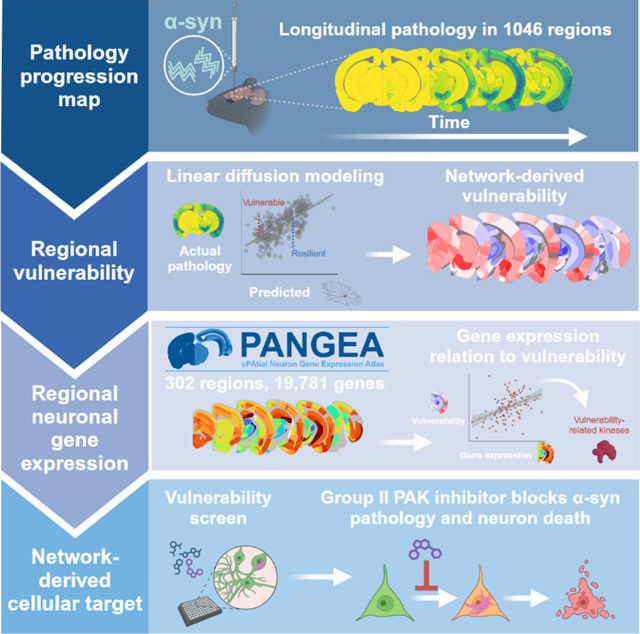

## INTRODUCTION

Parkinson’s disease (PD) is a progressive disorder characterized by loss of dopaminergic neurons and accumulation of α-synuclein Lewy pathology throughout the brain^1,2^. Extensive cellular dysfunction has been documented in PD as well as cell and animal models, with prominent involvement of mitochondria, lysosomes, and synapses^3^. However, PD manifests as a network disorder^4^. While dopaminergic neurons are lost in the substantia nigra pars compacta, movement symptoms are manifested due to disruption of basal ganglia control of motor cortical output. People with PD also exhibit a range of non-motor symptoms which are attributed to dysfunction in many other central and peripheral nervous system circuits^5^. α-Synuclein Lewy pathology is present in these disrupted circuits, and recent evidence suggests that misfolded α-synuclein may itself progress through synaptic connections, acting as the substrate of progressive dysfunction^5,6^.

Symptomatic therapies, such as dopamine replacement and deep brain stimulation, have sought to correct the disrupted networks in PD^4^. However, disease-modifying therapies have largely been identified and targeted towards cellular mechanisms^7^, with minimal information about how those therapies may influence the network at large. This is partially because genes disrupted in disease are easily related to their encoded proteins and the cellular function and dysfunction of those proteins can be assayed in high-throughput cellular assays. Another reason for the lack of network-level target identification is that it has been difficult to understand the network-level pathological disruptions that occur in PD when pathology can only be assessed at disease end-stage.

Neuroimaging has arisen as one method to assay longitudinal measurements of pathological outcomes. In addition to structural magnetic resonance imaging (MRI) and neurotransmitter receptor imaging, one can directly measure the regional residence of pathological proteins with positron emission tomography (PET) tracers. This technology currently exists for amyloid β and tau pathologies^8^, and a variety of mathematical models have been used to predict pathology patterns using anatomical connectivity, functional connectivity, or regional proximity to form the base network^9–23^.

While there are currently no PET tracers available for pathological α-synuclein^24^, we have leveraged the same network modeling to understand pathology progression in seed-based animal models. In these models, small amounts of misfolded α-synuclein or tau are injected into the brains of wildtype mice^25,26^. We have shown the subsequent induction of pathology is constrained by anatomical connectivity^27–30^. However, connectivity alone is not sufficient to predict pathology in all regions. Cell autonomous vulnerability likely underlies differences in model predictivity, and we can use the residual variance in a connectivity-based model to estimate regional vulnerability^31^.

In the current study, we employed a network-based approach to identify novel targets for PD, followed by cellular validation to confirm their potential. While we have previously established the ability to generate regional vulnerability measures using network modeling^27^, efforts to translate this into molecular insight were impinged by two main factors—low pathology resolution (134 brain regions) and limited regional gene expression data (4277 genes). We have now implemented a user-guided workflow to generate quantitative pathology measures in 1046 brain regions at 8 timepoints in mice injected with α-synuclein pre-formed fibrils. Regional pathology burden was well-described by linear diffusion through anatomical connections, and residuals in this model were used to develop a measure of relative regional vulnerability. We also developed a sPAtial Neuronal Gene Expression Atlas (PANGEA) with coverage of 19,781 genes in 302 of the 722 gray matter regions in the Allen Brain Atlas. We integrated regional vulnerability with PANGEA to identify gene expression patterns related to vulnerability. Interestingly, we identified gene sets associated with both resilience (oxidative phosphorylation) and vulnerability (complement/coagulation cascade) to developing pathology. By cross-referencing kinases associated with vulnerability to those whose expression was altered in neurons with Lewy pathology^32^, we identified 12 kinases with a possible association with cellular vulnerability. We then screened those kinases and identified p21-activated kinases (PAKs) as modifiers of α-synucleinopathy in primary neurons. We isolated this effect to group II PAKs and showed that group II PAK inhibition reduces α-synuclein pathology and rescues neuron death. The protective effect of PAK inhibition is present even if delivery is delayed until after pathology formation. Finally, we demonstrate that PAKs have a punctate distribution surrounding α-synuclein pathology, suggesting that they are actively involved in aggregate formation or clearance. Together, these results demonstrate the power of network-level analysis to reveal molecular insight and novel targets for therapeutic development in PD.

## RESULTS

### Spatiotemporal quantification of α-synuclein pathology at 8 timepoints in 1046 brain regions

**Figure 1.**
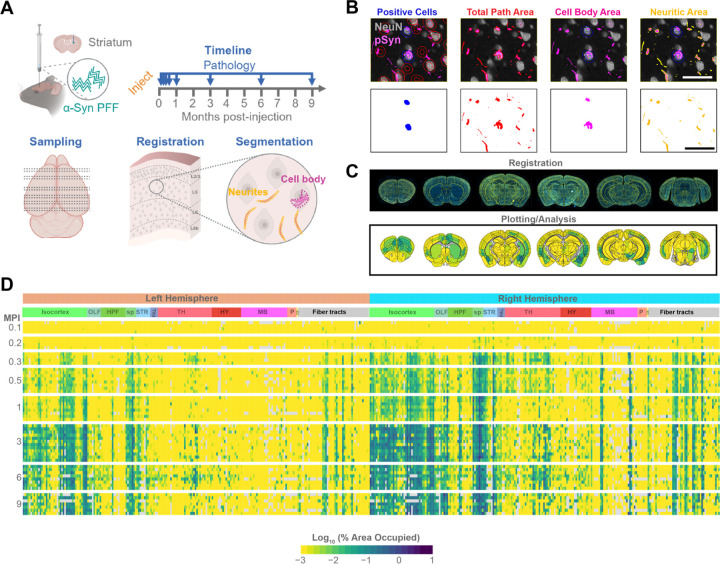
Spatiotemporal quantification of a-synuclein pathology at 8 timepoints in 1046 brain regions (A) Experimental schematic. Wildtype mice were injected in the dorsal striatum with α-synuclein pre-formed fibrils (PFFs). Mice were aged a further 0.1, 0.2, 0.3, 0.5, 1, 3, 6, or 9 months post-injection (MPI). Coronal sections representing the majority of regions with pathology were routinely sectioned for all mice. Sections were stained and registered to the Allen Brain Atlas (ABA) CCFv3 and multiple forms of pathology were segmented and quantified. (B) Example of pathology segmentation from mouse brain tissue stained for neurons (NeuN) and α-synuclein pathology (pSyn). The top panels show how the segmentation performs on the tissue while the lower panels are the resulting pathology masks. Scale bar = 50 μm. (C) Example set of sections registered to the ABA (top panel) and the resulting quantitative pathology plots (bottom panel). (D) Heatmap plot of all regional pathology measures from 0.1–9 MPI. Each row represents an individual mouse, and each column represents a brain region with major regional designations labeled at the top.

We have previously hypothesized that anatomical patterns of protein pathology arise from a combination of intrinsic cellular vulnerability and network connectivity^31^. To test this hypothesis, we needed to develop high-resolution maps of α-synuclein pathology progression over time, computational prediction of pathology based on anatomical connectivity, a brain-wide gene expression map, and cellular validation of network-derived vulnerability measures. To induce α-synuclein pathology, we injected 3-month-old wildtype mice with α-synuclein pre-formed fibrils (PFFs) in the dorsal striatum (Fig. 1A). Mice were aged 0.1, 0.2, 0.3, 0.5, 1, 3, 6, or 9 months post-injection (MPI) to capture the progression of pathology. Each brain was sampled systematically through coronal sectioning, registered to the Allen Brain Atlas (ABA) CCFv3 and segmented for multiple types of α-synuclein pathology.

α-Synuclein PFF injection induces broad neuritic and cell body inclusions positive for pS129 α-synuclein, similar to PD brains. While cell body inclusions represent the impact of pathology in a particular region, neuritic pathology could also be present in neurons projecting to or through a region. Therefore, following staining of tissue for pS129 α-synuclein and NeuN for neuronal cell bodies, we developed segmentation strategies to capture the number of neurons with cell body inclusions or the area of total, cell body, or neuritic pathology (Fig. 1B). Each section was registered to the ABA CCFv3 using a suite of software known as the QUINT workflow^33^ (Fig. S1A). We adapted this workflow to include our segmentation measures^34^ and developed an accompanying plotting and statistical analysis platform for registered datasets (Fig. 1C). We were able to map total, cell body, and neuritic α-synuclein pathology in 1046 regions of the brain at the 8 timepoints (Fig. 1D, S1B, S1C).

### α-Synuclein PFFs induce a broad pathology pattern, influenced by regional neuron types

**Figure 2.**
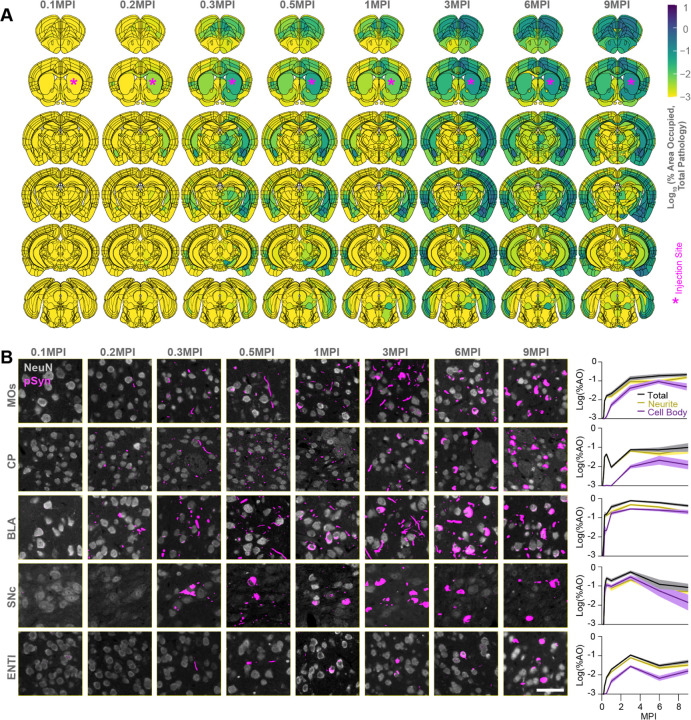
α-Synuclein PFFs induce a broad pathology pattern, influenced by regional neuron types (A) Pathology heatmaps representing the average pathology in each anatomical brain region at the corresponding timepoint. *Injection sites. (B) Representative α-synuclein pathology staining across timepoints for 5 different brain regions. Plots on the right represent the total, cell body, or neuritic pathology quantification for those regions. Scale bar = 50 μm.

Pathology develops rapidly with motor cortex and amygdala as early as 0.2 MPI (Fig. 2A, 2B). Despite direct proximity to the injection site, the caudoputamen is relatively slow to develop pathology. Even when pathology does develop there, it is typically neuritic. Robust cell body pathology does not develop in the caudoputamen until 3 MPI. In contrast, the substantia nigra pars compacta rapidly develops pathology, including cell body pathology. That pathology has peaked by 3 MPI, and later timepoints see a dramatic reduction of pathology. We have previously found that this pathology loss directly relates to neuron loss in that region^30^. Some of the differences in regional pathology development may relate to cell type vulnerability. Caudoputamen is largely composed of inhibitory medium spiny neurons, while the cortex has an abundant excitatory neuron population that bears the pathology, and the substantia nigra pars compacta is composed of dopaminergic neurons. Globally, pathology is initially more lateralized, with ipsilateral regions showing more pathology. Later, pathology becomes more broadly distributed bilaterally.

To understand if our coronal (2D) sampling strategy was able to capture brain-wide pathology patterns, one brain was fixed at 3 MPI, optically cleared, and stained for pS129 α-synuclein. The whole brain was imaged (3D) and registered to the ABA CCFv3. Segmentation of total pathology volume was performed as a direct comparison to pathology area captured in coronal sections (Fig. S2A, S2B). Of note, imaging quality was insufficient with this modality to confidently distinguish between cell body and neuritic inclusions, so only total inclusion area was measured. Overall, there was a good correlation between 2D and 3D datasets (Fig. S2C–S2F). However, there were some discrepancies between the two datasets that corresponded to major anatomical divisions. It was apparent that some of the over-representation in the 3D dataset was related to non-specific staining of ventricles, including those in the hypothalamus, and misregistration of regions (Fig. S2G, S2H). For example, the substantia nigra is a very thin nucleus in the ABA CCFv3 and the 3D registration misaligned the substantia nigra pars compacta, assigning the pathology in this region to other midbrain nuclei. Cortical laminar alignment was also sub-optimal in the 3D registration. We did identify some thalamic regions in the 3D dataset that were not well-sampled in the 2D dataset, partially because those nuclei are broadly distributed along the rostro-caudal axis and may not have been enriched in the assessed coronal samples (Fig. S2I, S2J). Overall, the comparison of 2D and 3D datasets highlighted the good coverage of the brain with coronal sampling, with more user control over accurate brain registration.

### Time- and region-dependent progression of α-synuclein pathology

**Figure 3.**
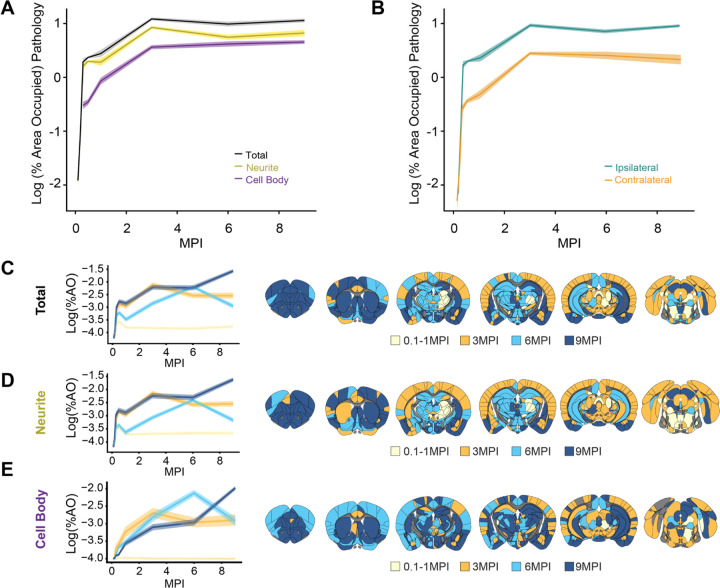
Time- and region-dependent progression of α-synuclein pathology (A) Plot of average pathology measured in all regions demonstrates the predominance of neuritic pathology, especially early, and the overall plateau of pathology at 3 MPI. (B) Plot of average pathology across hemispheres demonstrates the early predominance of ipsilateral pathology and later gains in contralateral pathology. (C) Total α-synuclein pathology peaks at different times in specific regional groups. The average pathology level of regions that peak at different times is plotted as a group in the panel on the left. The anatomical plots on the right display the peak time for each region. Similar data for neuritic (D) and cell body (E) α-synuclein pathology are also plotted.

After identifying interesting regional patterns of pathology, we sought to further assess global patterns of pathology development. We found that pathology is progressive up to 3 MPI, after which pathology plateaus (Fig. 3A). Neuritic pathology dominates early, with cell body pathology nearly catching up at later timepoints, when neuritic pathology is reduced. This later reduction of neuritic pathology may arise because neuritic pathology has consolidated into cell body inclusions, neurons cleared pathology, or neurons died. Ipsilateral pathology dominates at early timepoints, but pathology becomes distributed more bilaterally at later timepoints (Fig. 3B).

We next assessed the patterns of regional peak pathology levels. Generally, very few regions had peak pathology prior to 3 MPI (Fig. 3C–3E). As noted, many regions begin to peak at 3 MPI, and this is particularly true of total (Fig. 3C) and neuritic (Fig. 3D) pathology. Most caudal cortical regions, amygdala, and substantia nigra peak at 3 MPI, while rostral cortical regions, striatum, hippocampus, and some amygdala regions do not peak until 6 or 9 MPI. Similar patterns can also be seen for cell body pathology, albeit slightly delayed (Fig. 3E). Overall, the pathology in this model is progressive and relates to anatomical connectivity of the injection site, but with clear influence of regional vulnerability as well. To further assess these patterns and develop a measure of regional vulnerability, we turned to computational network modeling.

### Computational model of pathology based on anatomical connectivity shows predictivity of regional α-synuclein pathology

**Figure 4.**
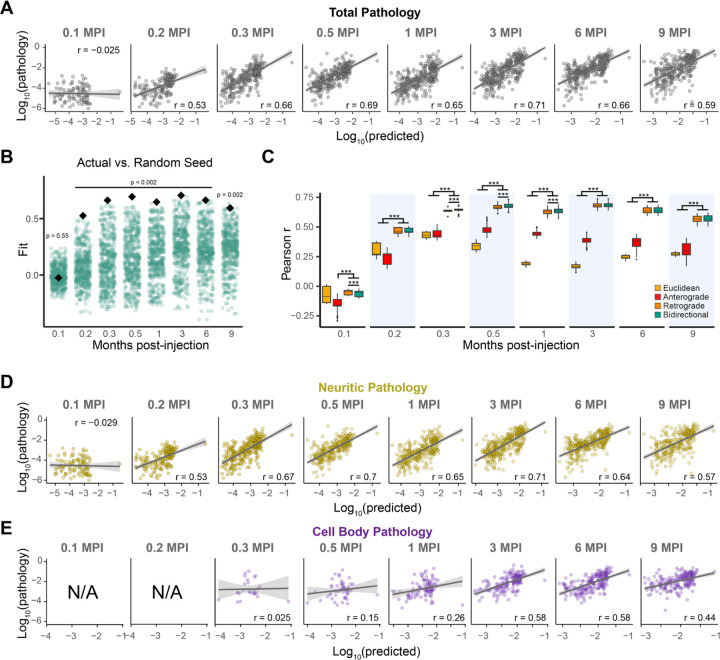
Computational model of pathology based on anatomical connectivity shows predictivity of regional α-synuclein pathology (A) Predictions of total α-synuclein pathology from linear diffusion models based on bidirectional (anterograde and retrograde) anatomical connections. Solid lines represent the line of best fit, and shading represents 95% confidence intervals. Each dot represents a different brain region where α-synuclein pathology was measured. (B) Comparison of Pearson’s *r* values obtained by fitting bidirectional spread models using actual (black diamond) and alternate (green circles) seed regions. (C) Comparison of Euclidean, anterograde, retrograde, and bidirectional model fits across 500 held-out samples derived from 500 iterations of a cross-validation process. Retrograde and bidirectional spread models performed significantly better than the anterograde spread model at all time points, and significantly better than the Euclidean spread model at all time points except 0.1 MPI (***P < 0.002). (D) Predictions of neuritic α-synuclein pathology from linear diffusion models based on bidirectional (anterograde and retrograde) anatomical connections. Solid lines represent the line of best fit, and shading represents 95% confidence intervals. (E) Predictions of cell body α-synuclein pathology from linear diffusion models based on bidirectional (anterograde and retrograde) anatomical connections. Solid lines represent the line of best fit, and shading represents 95% confidence intervals.

In previous studies, linear diffusion models have been shown to explain the spread of pathological tau and α-synuclein proteins along the brain’s structural connectome. These studies have determined that patterns of pathology occupancy are primarily driven by spread in the retrograde direction. Here, we build on this prior work by modeling the spread of pathology from high spatiotemporal resolution data, including both cell body and neuritic pathology. Consistent with our previous analysis with hundreds of annotated regions, the current analysis of higher resolution data yielded a high fit to actual pathology measures, as early as 0.2 MPI (Fig. 4A).

To evaluate model specificity to the injection site, we randomly selected 500 alternate seed regions and recomputed model fit using each of these regions. The experimental injection site resulted in a stronger correlation between actual and predicted pathology at every time point except the earliest (0.1 MPI), further supporting the specificity of the model fit to the connectivity from the striatum (Fig. 4B).

We statistically compared out-of-sample performance of our bidirectional diffusion model to models based on retrograde spread alone, anterograde spread alone, and Euclidean distance. The retrograde and bidirectional spread models performed significantly better than the Euclidean distance model at all time points except the first (0.1 MPI) and outperformed the anterograde spread model at every time point (Fig. 4C). The bidirectional spread model outperformed the retrograde spread model only slightly at 0.3, 0.6, and 1 MPI. These findings are consistent with earlier reports demonstrating that spread predominantly occurs in the retrograde direction.

Consistent with the observation that neuritic pathology dominates at early time points, we observed strong correlations between actual and model-predicted neurite pathology beginning at 0.2 MPI (Fig. 4D). In cell bodies, pathology spread was not evident until 0.3 MPI, and model fit remained weak until 3 MPI (Fig. 4E). This discrepancy likely arises from neuritic pathology developing rapidly while cell body pathology develops slowly, resulting in fewer regions with pathology to properly estimate model fit. In both cell bodies and neurites, the strongest fit was observed at 3 MPI. The fit of the actual site compared to alternate seed sites showed a corresponding performance, with good performance for neuritic pathology from 0.2 MPI onward (Fig. S4A), and a good fit for cell body pathology from 3 MPI onward (Fig. S4B).

Although linear diffusion model parameters were fit to a caudoputamen injection site, we explored the utility of this model in determining α-synuclein pathology progression following injection into other sites. Model parameters remained constant with those used for the caudoputamen injection site, but the initiation site was shifted throughout the rostro-caudal axis (Fig. S4C). Regions that have injections reported, including the olfactory bulb (MOB), cortex (MOs), hippocampus (CA1/CA3/DG), substantia nigra (SN), and pedunculopontine nucleus (PPN) show patterns similar to those previously reported^35–38^. Additional injection sites include the medial septum (MS/NDB), thalamus (VAL), and dentate nucleus of the cerebellum (DN). While these sites show what might be expected from spread through connectivity, they await experimental confirmation. For example, thalamic nuclei express low α-synuclein and therefore may be resistant to developing pathology.

Turning back to predictions from the caudoputamen injection site, we hypothesized that pathology progression is likely mediated by cell- and region-intrinsic factors including cell type and gene expression, in addition to anatomical connectivity. The differential vulnerability of anatomical regions can be observed if the predictivity of the model is plotted by anatomical region (Fig. S4D). Here, thalamic and mesencephalic regions can be seen to have lower pathology than expected by modeling. If we presume that local pathology is the result of a combination of anatomical connectivity and vulnerability factors, then regional vulnerability can be estimated as the difference between predicted and observed pathology (e.g., the residuals in the model fit to pathology data). We calculated residuals in the model at each timepoint and compared these values through a correlation matrix (Fig. S4D). Overall, regional residuals are well conserved across hemisphere, but residuals are not as consistent at early timepoints (0.1–0.5 MPI), likely due to the lower amount of pathology at those timepoints available to fit the model. Due to the high conservation of residuals across hemispheres and from 1 to 9 MPI, we averaged those values to create a composite regional vulnerability measure that could be used moving forward.

### Spatial neuronal gene expression atlas (PANGEA)

We hypothesized that the residuals of each region in the diffusion model (e.g., the variance not explained by connectivity) are explained, at least in part, by regional gene expression. To test this hypothesis, we aimed to directly compare the variance unexplained by connectivity to regional gene expression. For this, we needed a spatially resolved gene expression brain atlas. The first, and still one of the most widely used of these atlases is the ABA *in situ* hybridization data^39^. This massive resource is excellent for gene expression visualization but was not generated with the goal of regional gene quantification. While we and others have previously used this resource, only 4277 genes have expression patterns available after applying a standard quality control threshold^27,40^. A recent spatial transcriptomics atlas acquired with evenly-spaced spots covers a similar number of genes, but with quantitative infrastructure^41^. Other spatial transcriptomics technologies like MERFISH have enabled high-resolution transcript assignment, but only cover hundreds of genes^42^. Each of these atlases provides valuable insight into molecular classification of cells and anatomical regions. However, to optimally determine the relationship of gene expression and neuronal vulnerability to pathology, we required regional expression resolution, quantitative whole transcriptome coverage, and neuronal expression specificity.

**Figure 5.**
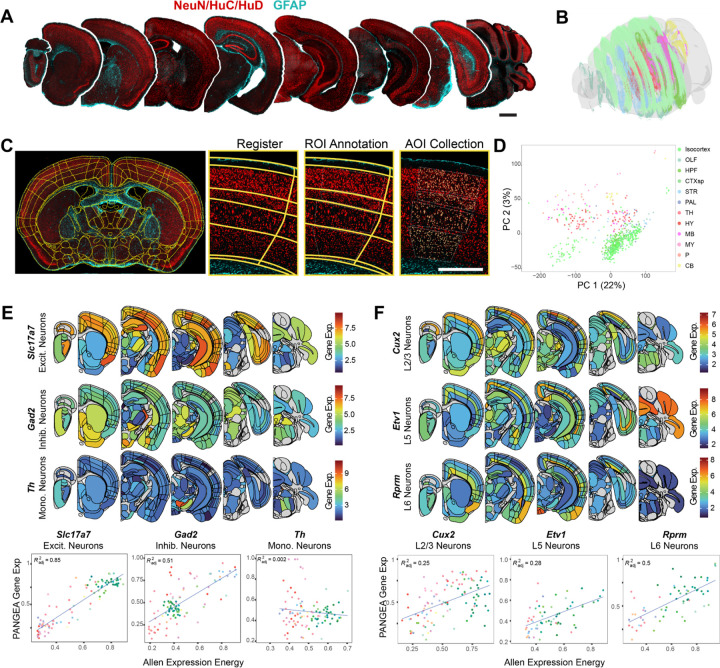
sPAtial Neuronal Gene Expression Atlas (PANGEA) (A) Representative coronal sections throughout the rostro-caudai axis of the mouse brain stained with NeuN/HuC/HuD (red) and GFAP (cyan). Scale bar = 1 mm. (B) Transparent 3D brain reconstruction of all sections sampled for this study placed in the appropriate 3D space. Made with MeshView. (C) Example segmented brain section on the GeoMx instrument. Subsequent panels demonstrate zoomed view of the registration, region-of-interest (ROI) annotation, and area-of-illumination (AOI) for the collection of regional neuronal transcripts. Scale bar = 0.5 mm. (D) Principal component analysis plot with each AOI plotted as a single dot colored by the major anatomical region. Regions show segregation mainly dependent on the major brain region. (E, F) Anatomical heatmaps and correlation plots comparing PANGEA data to Allen *in situ* hybridization expression energy data^1^. PANGEA gene expression related to neuron types shows the expected regional pattern from previous studies and correlates well with Allen expression energy, except for *Th*, which is very sparsely expressed. Correlation plot coloring is based on ABA anatomical region coloration (see legend on panel D).

We therefore developed the sPAtial Neuron Gene Expression Atlas (PANGEA) by performing GeoMx whole transcriptome atlas capture on segmented neurons in spatially registered brain sections (Fig. 5A, 5B). Coronal brain sections from 3 male and 4 female mice were stained with NeuN/HuC/HuD to label neuronal cell bodies and glial fibrillary acidic protein (GFAP) as a morphology marker. Brain sections were registered to the ABA CCFv3 using the QUINT workflow, and registrations were imported onto the GeoMx instrument to enable anatomically guided region-of-interest designation (Fig. 5C). The area of illumination for barcode collection and sequencing was based on segmentation of NeuN/HuC/HuD immunofluorescence. Thus, transcript barcodes covering the whole transcriptome were captured from neuronal soma in anatomically defined regions. Altogether, the atlas represents 302 of the 722 gray matter regions in the ABA CCFv3. Regions that were not sampled include cortical layer 1, which has few neurons, and hindbrain regions that are difficult to anatomically distinguish accurately. After quality control, the atlas covers the regional expression of 19,781 genes.

To discern general trends in the data, we performed a principal component analysis on all regions (Fig. 5D). Regions clustered within principal components 1 and 2 based on major brain divisions, with cortical regions separating from subcortical regions.

To discern general trends in the data, we performed a principal component analysis on all regions (Fig. 5D). Regions clustered within principal components 1 and 2 based on major brain divisions, with cortical regions separating from subcortical regions. To further examine the utility of this atlas, we examined the expression of known cell type and region markers, and then we compared these data to the ABA *in situ* hybridization atlas (Fig. 5E, 5F, S5). We found that the excitatory neuron marker *Slc17a7* is highly expressed in regions known to have excitatory projection neurons, while the expression of inhibitory neuron marker *Gad2* was lower in these regions and more highly expressed in subcortical regions (Fig. 5E). Regional expression of these genes also correlated well with the “expression energy” of the same genes in the ABA *in situ* hybridization atlas. In contrast, the catecholaminergic neuron marker *Th* was largely restricted to the substantia nigra, ventral tegmental area, and locus coeruleus, as expected. The regional correlation of *Th* to the ABA was low, likely due to its sparse expression. Known cortical layer markers also showed highly stereotyped regional expression across the neuraxis—*Cux2* in layer 2/3, *Etv1* in layer 5, *Rprm* in layer 6 (Fig. 5F). *Snca*, the gene encoding α-synuclein, shows high expression in cortex, especially layer 5, amygdala, hippocampus and substantia nigra pars compacta, and low expression in many thalamic and mesencephalic nuclei (Fig. S5A). This is consistent with previous evaluation of *Snca* mRNA expression and α-synuclein protein expression that had been mis-localized to the nucleus (Fig. S5B, S5C)^43^.

To further establish the utility of this dataset, we examine the brain-wide correlation of gene expression to the 4277 genes available in the ABA dataset. We found that PANGEA and the ABA data were positively correlated for the majority of genes (Pearson’s r mean=0.27641, minimum= −0.50310, maximum= 0.92024) (Fig. S5D).To determine the depth to which region-selective gene expression could be characterized with this method, we evaluated the expression of the top 10 genes previously identified as dopaminergic neuron selective using dopamine-driven RiboTRAP^44^ or enriched in the substantia nigra over the ventral tegmental area. We found that all 10 genes (*Chrna6, Cpne7, Ddc, En1, Gch1, Pitx3, Slc6a3, Slc10a4, Slc18a2*, and *Th*) were strongly enriched expression in the substantia nigra pars compacta in PANGEA (Fig. S5E).

These examples highlight that PANGEA may be used to validate previously established markers, but it may also be used to identify novel gene expression markers that distinguish nearby anatomical regions such as cortical layers. PANGEA data can be browsed and downloaded at https://lume.tv/PANGEA/.

### Regional vulnerability is associated with specific gene co-expression networks

**Figure 6.**
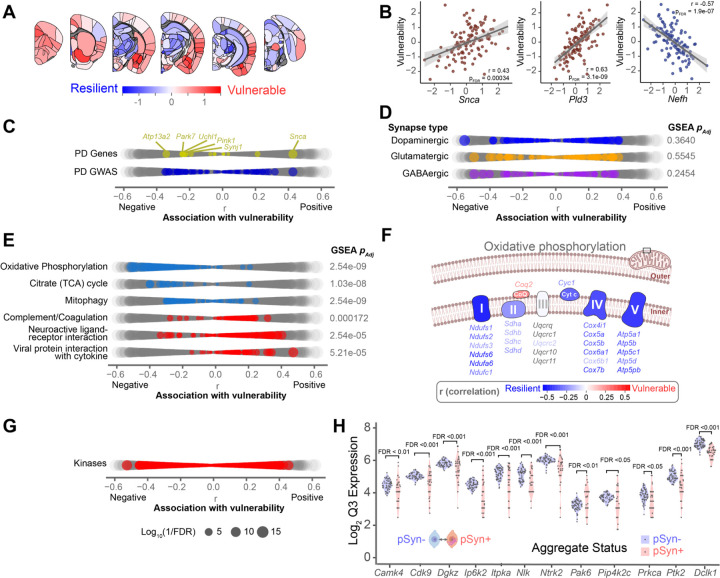
Regional vulnerability is associated with specific gene co-expression networks (A) Regional vulnerability heatmap plotting average region residuals (1,3,6,9 MPI) from the linear diffusion model of pathology spread. (B) Scatterplot of *Snca* and top genes associated with vulnerability from PANGEA (Pearson’s *r*). Each dot represents the expression and vulnerability score (residual) from each region in PANGEA. Solid lines represent the line of best fit, and shading represents 95% confidence intervals. (C) Dot plot highlighting causal PD genes, and GWAS identified PD genes. Dots are scaled according to the significance of the correlation between gene expression and regional vulnerability (FDR corrected *p*-value). (D) Dot plot highlighting synaptic genes associated with specific neuron subtypes. (E) Dot plot highlighting genes of top gene sets identified as differentially enriched by GSEA. (F) Schematic of the oxidative phosphorylation electron transport chain. Genes relevant to each component of the electron transport chain are colored by their association to regional pathology vulnerability. (G) Dot plot highlighting kinase genes. (H) Violin plots of the gene expression level of 12 kinases positively associated with regional pathology vulnerability that also showed differential expression between neurons with and without α-synuclein inclusions in the cortex of the α-synuclein PFF model^2^.

The goal of generating PANGEA was to determine if there are gene expression patterns that are related to regional vulnerability (Fig. 6A). We therefore determined the correlation of all gene expression patterns with the regional vulnerability to the development of α-synuclein pathology. As expected, *Snca* shows a positive correlation with vulnerability (Fig. 6B). However, *Snca* did not show the highest correlation with vulnerability or resilience of all genes, suggesting that there are gene expression patterns that have a higher influence on whether a region develops α-synuclein pathology, than the substrate gene itself. *Pld3* showed the highest correlation with vulnerability, while *Nefh* showed the most negative correlation (Fig. 6B).

Among causative and risk genes for PD, *Snca* showed the highest correlation with regional vulnerability (Fig. 6B, 6C). While *Snca* was the only PD causative gene with a significant positive correlation with vulnerability, several causative genes had a significant negative relationship with vulnerability (Fig. 6B). That is, they are more highly expressed in regions that are less likely to develop α-synuclein pathology. Mutations in all these genes (*Atp13a2, Park7, Uchl1, Pink1, Synj1*) are thought to lead to loss-of-function. The higher expression of these genes in regions that are less likely to develop aggregates suggests that their function may be protective in PD and that mutations, or lower expression, may lead to an increased propensity to develop pathology. None of the GWAS genes had a higher correlation with vulnerability than familial PD genes.

We next examined the relationship between vulnerability and known cell type markers, since excitatory and catecholaminergic neurons are thought to be more vulnerable to developing α-synuclein pathology (Fig. 6D). None of the gene sets relating to dopaminergic, excitatory, or inhibitory synapses were significantly enriched related to vulnerability. This could be due to the intrinsic mixture of cell types in many brain regions, or the redundancy of genes expressed in different synapse types.

We next aimed to identify gene pathways associated with vulnerability in an unbiased manner. We therefore performed gene set enrichment analysis to identify gene sets significantly enriched in either positive or negative association with vulnerability (Fig. S6). Overall, we found 70 gene sets that were significantly enriched for vulnerability or resilience to pathology (FDR<0.05). Mitochondrial oxidative phosphorylation, citrate cycle, and mitophagy were all highly over-represented in the resilient side (Fig. 6E, S6). Even at the individual gene level, oxidative phosphorylation genes are more highly expressed in regions that are more resilient to α-synuclein pathology (Fig. 6F). This increased expression could represent a conserved resilience pattern since mitochondrial toxins are known to relate to PD. Thus, regions with lower expression of these pathways intrinsically may be more susceptible to toxicity induced by mitochondrial perturbation, including by α-synuclein pathology^32^. Gene patterns positively related to vulnerability included the complement and coagulation cascade, neuroactive ligand-receptor interaction, and viral protein interaction with cytokine (Fig. 6E, S6). The complement and coagulation cascade is a key process in innate immunity, regulating the body’s response to foreign pathogens. The positive relationship between this cascade and vulnerability suggests higher regional expression of these genes relates to increased regional vulnerability to pathology.

While each of these associations provides evidence for pathways involved in the development of pathology in PD, many are difficult to manipulate due to their essential nature or lack of druggable targets. We therefore focused on a class of genes—kinases—that are amenable to manipulation to directly assess whether network-derived vulnerability can be directly related to cellular vulnerability and novel therapeutic targets. While kinases as a class showed no directional enrichment related to vulnerability, we postulated that kinases enriched in vulnerable regions would make good targets for inhibition. We identified 106 kinases whose expression was significantly related to vulnerability (Pearson’s *r*, FDR<0.05) (Fig. 6G). To further narrow this list, we only included genes that we had previously identified as significantly different between α-synuclein inclusion-bearing neurons and their neighbors^32^. The final list included 12 kinases (*Cdk9, Nlk, Pak6, Dclk1, Prkca, Ptk2, Ntrk2, Ip6k2, Camk4, Itpka, Pip4k2c*, and *Dgkz*) that we hypothesized had a direct relationship with regional neuronal vulnerability to developing α-synuclein pathology (Fig. 6H).

### PAK inhibition is protective in a neuronal model of α-synucleinopathy

**Figure 7.**
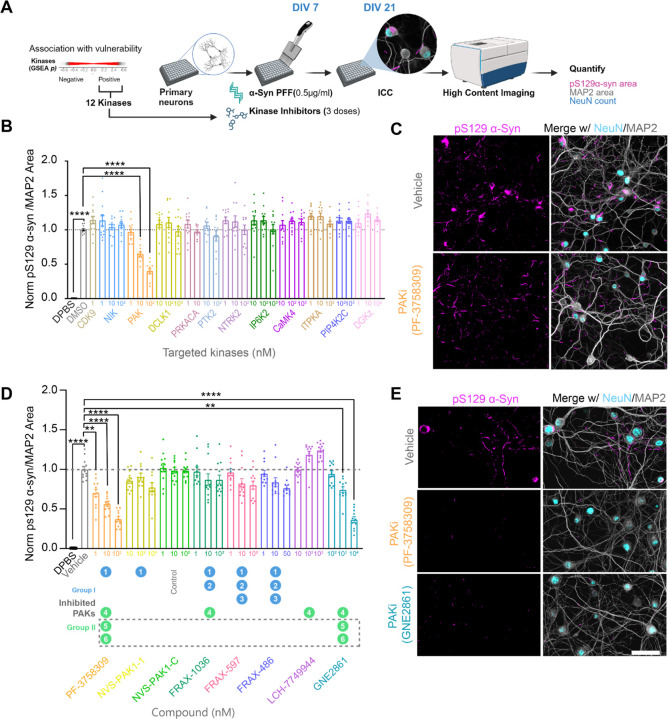
PAK inhibition is protective in a neuronal model of α-synucleinopathy (A) Schematic for kinase inhibitor screen. (B) Kinases were inhibited at 3 different doses in primary hippocampal neurons treated with α-synuclein PFFs and pathology was quantified relative to MAP2 area. Data presented as mean ± SEM with individual values plotted. N=12 independent wells from 4 separate cultures, *p*-values represent fold-change compared to vehicle control with Welch ANOVA test and Dunnett’s T3 multiple comparison test: ****p < 0.0001. (C) Representative image of pS129 α-synuclein in vehicle treated neurons compared to neurons treated with 100 nM of PAK inhibitor. (D) PAK inhibitors with different selectivity were used at 3 different doses in primary hippocampal neurons treated with α-synuclein PFFs and pathology was quantified relative to MAP2 area. Data presented as mean ± SEM with individual values plotted. N=12 independent wells from 4 separate cultures, *p*-values represent fold-change compared to vehicle control with Welch ANOVA test and Dunnett’s T3 multiple comparison test: **p < 0.001, ****p < 0.0001. (E) Representative images of pS129 α-synuclein in vehicle treated neurons compared to neurons treated with the highest dose of PF-3758309 or GNE2861. Scale bars = 50 μm.

To evaluate the role of these 12 kinases at the cellular level, we performed a kinase inhibitor screen in a primary neuron model of α-synucleinopathy. Primary hippocampal neurons were cultured and transduced with α-synuclein PFF at 7 days *in vitro* (DIV) to induce pathology (Fig. 7A). On the same day, neurons were treated with kinase inhibitors or vehicle control. Neurons were treated with kinase inhibitors at three doses spanning above and below their reported IC50 values. Neurons were fixed 14 days after treatment and stained for NeuN, MAP2, and pS129 α-synuclein to evaluate the effect of the test compounds on neuronal health and α-synuclein pathology. Some compounds induced neuron death (Fig. S7A, S7B), which could confound measurements of pathology. Therefore, treatments which caused more than a 25% reduction in NeuN count or MAP2 area were omitted from further analysis. Pathology was quantified as the pS129 α-synuclein positive area normalized to MAP2 area. The PAK inhibitor PF-3758309 showed a dose-dependent reduction of α-synuclein pathology where the maximum tested dose of 100 nM reduced pathology by around 50%. (Fig. 7B, 7C). No other tested compound showed a significant reduction in α-synuclein pathology (Fig. 7B, S7E).

The PAK family is composed of six members, which are categorized into two groups: Group I (PAK1, PAK2, and PAK3) and Group II (PAK4, PAK5, and PAK6). Since PF-3758309 inhibits PAK1 from group I and PAKs 4,5 and 6 from group II^45^, we aimed to narrow down the specific PAKs contributing to α-synuclein pathology. We used a suite of PAK inhibitors with differential specificity towards the PAK family (Fig. 7D, 7E). We did not observe a significant reduction of α-synuclein pathology with NVS-PAK1-1, a highly specific PAK1 inhibitor, as compared to its control; NVS-PAK1-C. Other PAK inhibitors like FRAX-1036 (inhibiting PAK1,2,4); FRAX-597 (inhibiting PAK1,2,3); FRAX-486 (inhibiting PAK1,2,3) also did not reduce α-synuclein pathology. We saw a trend of increased α-synuclein pathology with LCH-7749944, a PAK4 specific inhibitor, which is consistent with a previous study^46^. One additional compound, GNE-2861, which is specific for Group II PAKs, demonstrated a dose-dependent reduction in pathology, with approximately 50% reduction observed at the highest tested dose of 10 μM (Fig. 7D). Only PF-3758309 and GNE-2861 were able to reduce α-synuclein pathology, while PAK1 and PAK4 inhibitors did not, suggesting that the inhibition of PAK5 and/or PAK6 is likely responsible for the observed reduction in pathology (Fig. 7D, 7E). The doses at which PAK inhibitors were tested were well tolerated and did not induce significant cell death (Fig S7C, S7D).

*Pak4* knockout in mice is embryonic lethal around E11 due to cardiac abnormalities. These mice also show abnormalities in spinal cord motor neurons, axonal outgrowth, and thin neuroepithelium^47^. In contrast, *Pak5* knockout and *Pak6* knockout mice are viable and fertile and do not show any morphological defects in the brain. Both PAK5 and PAK6 show predominant brain expression, with lower expression levels observed in peripheral tissues^48,49^. These data suggest that PAK5 and PAK6 could be viable targets for further therapeutic development.

### Group II PAK inhibition is neuroprotective even with delayed treatment

**Figure 8.**
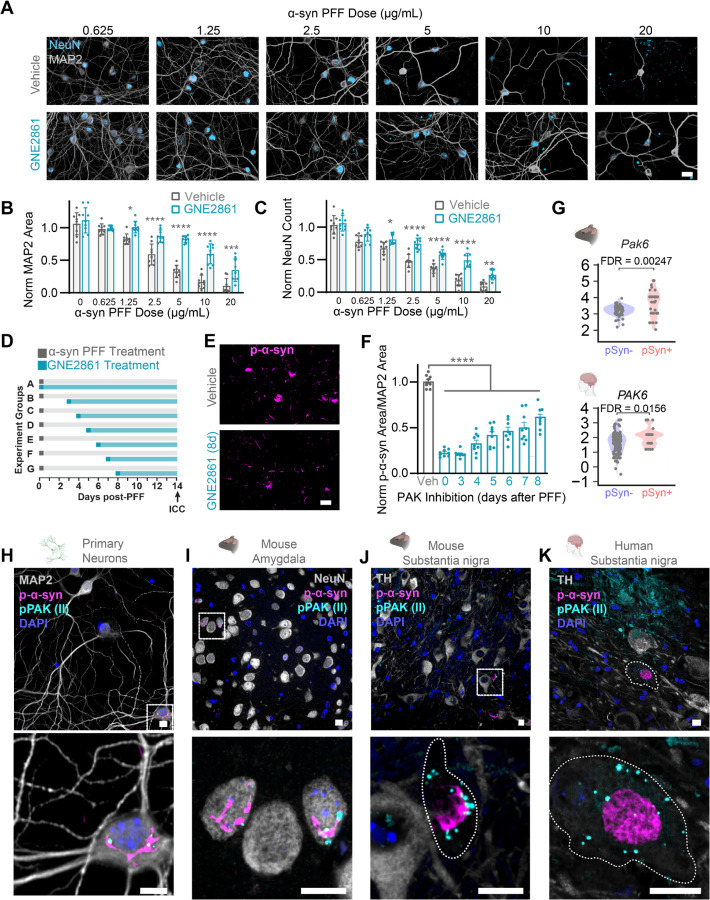
Group II PAK inhibition is neuroprotective even with delayed treatment (A) Representative images of primary neurons treated with increasing doses of α-synuclein PFFs. Scale bar = 20 μm. Both somatodendritic marker MAP2 (B) and neuronal nuclei marker NeuN (C) show dose-dependent reductions and are rescued by treatment with GNE2861. N=9 independent wells from 3 separate cultures. Data presented as mean ± SEM with individual values plotted, *p*-values represent fold-change compared to vehicle control with 2-way ANOVA test with Sidak’s multiple comparison test, *p <0.05, ***p <0.01, ***p = 0.001, ****p < 0.0001. Scale bar = 20 μm. (D) Schematic of the delayed treatment experiment. (E) Representative images of pS129 α-synuclein pathology after 8-day delayed treatment of 10 μM GNE2861. Scale bar = 20 μm. (F) Quantification of α-synuclein pathology in neurons following GNE2861 treatment at different intervals after α-synuclein PFF treatment. N=9 independent wells from 3 separate cultures, *p*-values represent fold-change compared to vehicle control with Welch ANOVA test and Dunnett’s T3 multiple comparison test: ****p < 0.0001. (G) Quantitation of PAK6 RNA in α-synuclein inclusion-bearing neurons and non-inclusion-bearing neurons within the same PD brain and in α-synuclein PFF injected mice. (H) Representative images for co-immunofluorescence of α-synuclein inclusions and pPAK (group II) that shows punctate distribution of PAKs near inclusions in primary neurons, (I) mouse amygdala, (J) substantia nigra, and (K) human PD substantia nigra. TH = tyrosine hydroxylase. Scale bars = 10 μm.

We aimed to further assess the protective effect of group II PAK inhibition. GNE2861 was selected as the lead compound due to its specificity for group II PAKs. In primary neurons, neuronal death is not induced by the α-synuclein PFF dose (0.5 μg/mL) over 21 DIV, as previously used. We therefore treated neurons with increasing doses of α-synuclein PFFs (0.625 to 20 ug/mL) and aged neurons to 28 DIV. This treatment paradigm induced a significant dose-dependent reduction of MAP2 area (Fig. 8A, 8B) and neuron count (Fig. 8A, 8C). Treating neurons with 10 μM of GNE2861 was able to rescue the reduction of NeuN count and MAP2 area at each tested dose (Fig. 8A, 8B, 8C). compound due to its specificity for group II PAKs. In primary neurons, neuronal death is not induced by the α-synuclein PFF dose (0.5 μg/mL) over 21 DIV, as previously used. We therefore treated neurons with increasing doses of α-synuclein PFFs (0.625 to 20 ug/mL) and aged neurons to 28 DIV. This treatment paradigm induced a significant dose-dependent reduction of MAP2 area (Fig. 8A, 8B) and neuron count (Fig. 8A, 8C). Treating neurons with 10 μM of GNE2861 was able to rescue the reduction of NeuN count and MAP2 area at each tested dose (Fig. 8A, 8B, 8C).

There are several potential mechanisms to reduce pathology and rescue neuron health—reduction of α-synuclein PFF internalization, blocked escape of PFFs from the lysosome, decreased α-synuclein monomer recruitment, increased degradation of fully-formed aggregates, etc. Treating neurons with inhibitors at the same time as α-synuclein PFF administration does not enable us to distinguish these mechanisms. To gain insight into how group II PAK inhibition reduces pathology and rescues neuron health, neurons were treated with the PAK inhibitor 0, 3, 4, 5, 6, 7, or 8 days after α-synuclein PFF treatment (Fig 8D). Remarkably, GNE-2861 treatment reduced α-synuclein pathology even when administered up to 8 days after PFF treatment (Fig. 8E, 8F). α-Synuclein PFFs are fully internalized within 24 hours of treatment^50^, so the fact that treatment with inhibitor reduces pathology even 8 days after α-synuclein PFF administration suggests that group II PAK inhibition does not act on the initial steps of internalization or lysosomal escape, but rather on cytosolic aggregate formation or clearance.

We previously found increased expression of *PAK6* mRNA in α-synuclein inclusion-bearing neurons when compared to non-inclusion-bearing neurons within the same PD brain and in α-synuclein PFF injected mice^32^ (Fig. 8G). Together, this provides evidence that *PAK6* transcript is upregulated in neurons with α-synuclein inclusions. To further understand the relationship of PAK protein to inclusion-bearing neurons, we stained primary neurons, mice, and human brain tissue with a phospho-PAK antibody selective for group II PAKs together with α-synuclein pathology. The phosphorylation site recognized by the antibody is at an activating autophosphorylation site in PAKs^51^. We observed distinct granular intraneuronal phospho-PAK puncta only in neurons with α-synuclein inclusions in primary hippocampal neurons (Fig. 8H, S8A) and in mouse amygdala (Fig. 8I) and substantia nigra (Fig. 8J) 3 MPI. These puncta were directly adjacent to pS129 α-synuclein inclusions, suggesting a potential physical interaction. These distinct puncta were not present in neurons without α-synuclein inclusions or in cultures treated with PBS (Fig. S8B) or monomer injected mice (Fig. S8C). We also stained substantia nigra tissue from postmortem human PD brains (Fig. 8K). We observed phospho-PAK puncta in the Lewy body-bearing neurons in TH-positive cells, however their distribution was throughout the soma and was not restricted to areas with Lewy bodies. Together, these studies suggest that group II PAKs are elevated and activated in neurons with α-synuclein inclusions, and group II PAK inhibition may be a viable therapeutic approach for PD and related α-synucleinopathies.

## DISCUSSION

In the current study, we aimed to bridge the gap between network and cellular vulnerability to α-synuclein pathology. To reach this goal, we undertook four related projects: 1) Seed-induced cell body and neuritic α-synuclein pathology was mapped from 0.1 to 9 months after injection in 1046 brain regions of 55 mice. 2) Linear diffusion models were developed to assess the relationship of pathology to anatomical connectivity networks and to develop relative measures of regional vulnerability. 3) Neuronal gene expression was assessed regionally throughout the brain (PANGEA), enabling access to whole genome expression data for our work and others. 4) The comparison of gene expression to regional vulnerability enabled the identification and validation of group II PAKs as potential therapeutic targets for PD. To date, there are no disease-modifying therapies for PD. We propose that a network-to-cell approach could be a viable strategy for the identification and validation of disease-modifying therapies. In addition to validation of this strategy in this pathology model, we also identified several new aspects of disease and brain biology that we explore further below.

Our study provides an extensive examination of cell body and neuritic pathology in the mouse brain following injection of α-synuclein PFFs in the dorsal striatum. While we and others have previously mapped α-synuclein pathology in this model^29,30,52^, the current study extends previous studies primarily in terms of resolution. Our previous work focused on total pathology in 172 brain regions. Here, advanced cell segmentation and brain registration were used to map total, cell body, and neuritic pathology in 1046 brain regions, including cortical laminar distribution. This map was assembled using coronal sections through the mouse brain. We explored the alternative strategy of mapping pathology in a cleared whole brain to ensure a full examination of pathology. In comparing three- and two-dimensional datasets, we found that they were largely correlated, suggesting we have captured the majority of data present in the brain with coronal sections. However, we also found that our three-dimensional dataset misrepresented pathology in some regions due to insufficiently accurate registration and non-specific ventricular staining. Nevertheless, three-dimensional analysis also enabled us to identify a sparse population of cells in thalamic nuclei that develop pathology and was missed in our coronal sectioning. Altogether both two- and three-dimensional analyses capture overall pathology patterns and specific experimental requirements should dictate the method used.

Clear patterns emerged in our pathology analysis. Pathology develops rapidly, with consistent and extensive pathology as early as 9 days after injection. Pathology is initially more lateralized towards the ipsilateral hemisphere and becomes more bilateral at later time points. Pathology has peaked in many, but not all regions, by 3 MPI. Many cortical regions, especially, have peaked by 3 MPI, while subcortical regions such as the striatum, hippocampus, thalamus, and some amygdala regions continue to develop pathology at later timepoints. There is also a distinction in the type of pathology that develops. Neuritic pathology precedes cell body pathology, although cell body pathology shows similar, but delayed, patterns. Eventually, cell body pathology catches up, but even at 9 MPI, neuritic pathology dominates. This shift from neuritic pathology to cell body pathology is likely similar to the shift in aggregate size distribution described by Dadgar-Kiani and colleagues^52^. These dynamics following a single bolus injection of α-synuclein PFFs could inform pathology analysis in human samples. While neuritic pathology also often dominates in PD, it may be useful to quantify both neuritic and cell body pathology as a relative measure of maturity of pathology in a particular region.

The computational model used in this study is similar to previous studies in that linear diffusion from the injection site along the anatomical connectome is used to estimate regional pathology burden. We had previously demonstrated that retrograde connectivity is better than anterograde connectivity or proximity at estimating pathology burden^30^. We confirm that relationship here and extend it by using a bi-directional linear diffusion model that allows for anterograde and retrograde progression simultaneously. This model performs only modestly better than retrograde connectivity alone, suggesting that most progression is retrograde. As more imaging ligands become available for assessing pathology or surrogate measures in human brain, the value of computational modeling of those brain-wide patterns increases. Recent studies modeling tau and amyloid β enabled by PET imaging ligands^17^ demonstrated that intracellular tau pathology also appears to progress through anatomical networks. Amyloid β pathology shows more relation to proximity, which would be expected for an extracellular pathology. In humans, computational modeling has been used to predict the site of pathology initiation, identify subtypes of disease, and develop individualized pathological prognosis^20^. It has also been suggested and demonstrated that regional gene expression patterns also relate to pathology progression patterns^15,18^. It therefore seems probable that anatomical connectivity and gene expression networks are two of the major underlying constraints on pathology progression, and we can use network predictions based on connectivity to mathematically derive gene expression patterns relevant to regional pathology development. Using this rationale, we calculated a regional vulnerability measure based on variation in the pathology data that was not explained by diffusion through anatomical connectivity.

In the α-synuclein PFF model, and in PD, most of the cells that develop α-synuclein pathology are neurons. We therefore sought a genome-wide, anatomically-resolved atlas of neuronal gene expression. Many brain transcriptome maps have been developed^39,41,42,53–56^ with more becoming available every year. Despite this, there was not a mouse brain transcriptome map that met the needs for this project. Therefore, we develop PANGEA, an anatomically resolved atlas covering 302 of the 722 gray matter regions with 19,781 genes above quality control thresholds. This atlas complements, but does not replace, the many other valuable atlases that are available. To make this dataset as accessible as possible, we developed an interactive web browser to display, analyze, and download the data: https://lume.tv/PANGEA/. PANGEA recapitulates many known neuronal gene expression patterns and should be useful to identify previously-unknown gene expression patterns related to anatomical distribution. During creation of the atlas, it was noted that NeuN does not stain many subcortical neurons as strongly as cortical neurons. Therefore, for subcortical regions, NeuN was combined with an antibody for HuC/HuD to enhance detection and collection of neuronal transcripts. The quality of this collection is reflected in the plotting of transcripts selective for dopamine neurons collected by Kilfeather and colleagues^44^. While PANGEA was best suited for our purposes, it is likely that additional transcript maps could also prove useful. For example, some of the vulnerability-related transcripts likely relate to cell types within that region, but cell type data is not available in PANGEA. Based on how slowly the caudoputamen injection site develops pathology, we presume that regions with more excitatory neurons are more likely to be vulnerable than those with inhibitory neurons, but atlases with comprehensive maps of cell type would be better positioned to parse out and compare cell type vulnerability. It is also possible that non-cell autonomous vulnerability is at play and non-neuronal gene expression patterns may relate to vulnerability. Again, this would be best assessed with alternate atlases.

Regional vulnerability measures derived from computational modeling were compared to regional gene expression patterns from PANGEA. There was a remarkably even distribution of gene expression correlations with vulnerability. The highest (*Pld3*) and lowest (*Nefh*) gene correlations were equidistant from the mean. As expected, *Snca*, the gene encoding α-synuclein, was positively correlated with vulnerability, but there were also surprising gene sets correlated or anti-correlated with vulnerability. Several PD genes were significantly anti-correlated with vulnerability (*Atp13a2, Park7, Pink1, Synj1*). Mutations in these genes in PD are thought to lead to loss-of-function. The fact that they are more highly expressed in regions that are less likely to develop pathology suggests that even lower gene expression in the absence of mutations may increase vulnerability to developing pathology. GSEA revealed clear pathways related to regional vulnerability. The pathways most negatively correlated with vulnerability were related to mitochondrial metabolism (oxidative phosphorylation, TCA cycle, mitophagy), suggesting that baseline metabolic capacity may be protective in α-synucleinopathies. Several familial PD genes are also involved in mitochondrial function including the previously mentioned *Park7*, and *Pink1*^57^, and even a body-wide reduction in mitochondrial complex I function has been linked to PD susceptibility^58^.

Patterns positively related with vulnerability largely related to complement and cytokine related gene expression. The complement/coagulation cascade has shown to be elevated in A53T α-synuclein transgenic mice^59^, α-synuclein PFF-injected rats^60^, and PFF-injected mice^32,59^, as well as the brains of people with PD^61–64^. A recent study demonstrated that α-synuclein aggregates lead to complement C3 activation in astrocytes and enhanced C3 receptor activation and apoptosis in nearby neurons^65^. Therefore, a higher baseline expression of complement and coagulation genes may increase intrinsic vulnerability to α-synuclein-induced toxicity.

While mitochondrial metabolism and complement and cytokine pathways are interesting from a biological perspective, they are also difficult to target directly either for mechanistic assessment or therapeutic development. Therefore, we focused on kinases that were positively associated with vulnerability with the rational that those kinases are expressed more highly in regions that develop more pathology and therefore may be amenable to targeting with kinase inhibitors. To further narrow this list, we also filtered by kinases that were differentially expressed in neurons bearing α-synuclein inclusions^32^. After filtering, we identified 12 kinases related to regional vulnerability and differentially-regulated in inclusion-bearing neurons to screen for modification of pathology in neurons. Some of these kinases were previously associated with α-synucleinopathies. Doublecortin-like kinase 1 (DCLK1) has been shown to regulate α-synuclein levels, directly implicating it in susceptibility to PD^66^. Inositol hexaphosphate kinase 2 (IP6K2) is reported to be neuroprotective, and its loss leads to PINK1-mediated increased mitochondrial fission and mitophagy^67^. Inositol-Trisphosphate 3-Kinase A (ITPK-A) has not been associated with α-synucleinopathy however, ITPK-B has been correlated with higher expression of α-synuclein^68^ and shown to protect against α-synuclein aggregation^69^.

Out of 12 kinases, only inhibition of PAKs had a clear, protective effect on neurons. Further screening showed that this effect is related to inhibition of PAK5/6. Group II PAK inhibition not only reduced pathology, but reduced neuron death, and proved protective even when administered up to 8 days after α-synuclein PFF addition. PAKs are serine/threonine kinases and based on their structure and sequence homologies are classified into two groups. Group I consists of PAK1, PAK2, and PAK3, and group II consists of PAK4, PAK5, and PAK6. Both subgroups share some functions while also having distinct roles, capable of participating in various intracellular signaling pathways^70,71^. Studies of group I PAK function highlight roles in actin cytoskeletal dynamics, apoptosis, and regulation of gene transcription, mostly in cancer^72,73^. PAK5 and PAK6 have been implicated in presynaptic vesicle dynamics by phosphorylating pacsin1 and synaptojanin1^74,75^. Both these proteins appear to mediate postsynaptic receptor endocytosis and presynaptic vesicle uncoating^76^. PAKs show differential expression pattern from fetal brain to adult brain, both in mice and humans^77^. PAK6 is co-expressed with androgen receptor and glucocorticoid receptors in dopaminergic regions of the brain, suggesting it may play a role in modulating dopaminergic neurotransmission through steroid hormones^78^. The similar expression pattern of both PAK5 and PAK6 in the brain and no gross deficit observed when either *Pak5* or *Pak6* is knocked out in mice suggests functional redundancy between these two proteins^49,79^.

Specific inhibitors for PAK5 or PAK6 are not available, so it was not possible to determine whether the observed effect is mediated through PAK5 and/or PAK6. Interestingly, PAK6 has been shown to interact with PD protein leucine-rich repeat kinase 2 (LRRK2). PAK6 controls neurite outgrowth and ciliogenesis, and this regulation is dependent on LRRK2^80,81^. Consistent with our study, a previous study also found elevated pS560 PAK in idiopathic and in *LRRK2*-PD brain^80^. Interestingly, phospho-*group I* PAK puncta were observed in AD brains suggesting a mechanistic division between the two diseases along PAK groups^82^. Further investigation is needed to determine the nature of these puncta, their potential localization within specific soma structures, and their role in α-synucleinopathy.

This study has several limitations. One limitation is the resolution of the connectivity data, and by extension the computational modeling and gene expression relationships. Current whole-brain connectivity maps are only available at mesoscale resolution, so cortical layers are not resolved. We have collected pathology and gene expression data with full regional resolution with the hope that future anatomical connectivity maps will have cortical laminar resolution and can be used to improve the specificity of analysis. The limitations of coronal sampling compared to full three-dimensional reconstruction are discussed above. For the current study, this form of sampling is optimal, but the comparison of two- and three-dimensional data in the future may enhance knowledge of pathology progression. The computational model used in this study is also simple. This is both a strength and a weakness. The strength is that anatomical connectivity alone provides strong predictivity and the model’s simplicity contributes to its interpretability and computational efficiency. The weakness is that we may be missing some contributors to variation, such as neuron death, that are incorrectly interpreted as cellular vulnerability. Future models incorporating non-linear dynamics and other factors, such as aggregation rate and neuron death rate, may improve predictivity. Finally, our regional vulnerability maps are based on a single injection site. This injection site was chosen because mice develop pathology broadly in disease-relevant circuits such as the nigro-striatal and cortico-cortical circuits. However, the broad applicability of this vulnerability map to alternative injection sites in the future would be beneficial.

Overall, our study provides a proof-of-principle for the ability to translate network level findings related to pathology progression to cellular targets for therapeutic intervention in PD. We also provide several resources for the neuroscience and neurodegeneration community to study pathology and gene expression patterns. Finally, we identify a novel neuroprotective class of kinase inhibitors for further therapeutic development in PD.

## MATERIALS AND METHODS

### Animals

All housing, breeding, and procedures were performed according to the NIH Guide for the Care and Use of Experimental Animals and approved by the Van Andel Institute Institutional Animal Care and Use Committee (IACUC). *Mus Musculus* CD1 (strain 022; RRID: IMSR_CRL:022) mice were obtained from Charles River and used for primary neuron culture. Wild type C57BL/6J mice (000664; RRID: IMSRJAX:000664) were purchased from the Jackson Laboratory. Animals were kept on 12 h light/dark cycles, with food pellets and water *ad libitum*.

### α-synuclein PFF

Purification of recombinant mouse α-synuclein and generation of α-synuclein PFFs was conducted as described elsewhere^83–85^. The pRK172 plasmid containing the gene of interest was transformed into BL21 (DE3) RIL-competent *E. coli* (Agilent Technologies Cat#230245). A single colony from this transformation was expanded in Terrific Broth (12 g/L of Bacto-tryptone, 24 g/L of yeast extract 4% (vol/vol) glycerol, 17 mM KH2PO4, and 72 mM K2HPO4) with ampicillin. Bacterial pellets from the growth were sonicated and the sample was boiled to precipitate undesired proteins. The supernatant was dialyzed with 10 mM Tris, pH 7.6, 50 mM NaCl, 1 mM EDTA, and 1 mM phenylmethylsulfonyl fluoride (PMSF) overnight. Protein was filtered with a 0.22 μm filter and concentrated using Amicon Ultra-15 centrifugal filter units (Millipore Sigma Cat#UFC901008). Protein was then loaded onto a Cytiva HiLoad 26/600 Superdex 200 pg (Cytiva Cat#28989336) and 5 mL fractions were collected. Fractions were run on SDS-PAGE and stained with InstaBlue protein stain (ApexBio B8226) to select fractions that were highly enriched in α-synuclein. These fractions were combined and dialyzed in 10 mM Tris, pH 7.6, 50 mM NaCl, 1 mM EDTA, 1 mM PMSF overnight. Dialyzed fractions were applied to the MonoQ column (Cytiva, HiTrap Q HP 17115401) and run using a linear gradient from 25 mM NaCl to 1 M NaCl. Collected fractions were run on SDS-PAGE and stained with InstaBlue protein stain. Fractions that were highly enriched in α-synuclein were collected and dialyzed into DPBS. Protein was filtered through a 0.22 μm filter and concentrated to 7 mg/mL (α-synuclein) with Amicon Ultra-15 centrifugal filter units. Monomer was aliquoted and frozen at −80°C. For preparation of α-synuclein PFFs, α-synuclein monomer was shaken at 1,000 rpm at 37 °C for 7 days. Conversion to PFFs was validated by sedimentation at 100,000 × *g* for 60 minutes and by thioflavin S staining.

### Stereotaxic Injection

All surgery experiments were performed in accordance with protocols approved by the IACUC of Van Andel Institute. α-Synuclein PFFs were vortexed and diluted with DPBS to 2 mg/mL and sonicated in a cooled bath sonicator at 9°C (Diagenode Bioruptor^®^ Pico; 10 cycles; setting medium; 30 seconds on, 30 seconds off). 3–4 month old mice were deeply anesthetized with isoflurane and injected unilaterally into the right forebrain targeting the dorsal striatum (coordinates: +0.2 mm relative to Bregma, +2.0 mm from midline, −2.5 mm beneath the dura) Injections were performed using a 10 μL syringe (Hamilton 7635–01, NV) with a 34-gauge needle (Hamilton 207434, NV) injecting 5 μg α-synuclein PFFs (2.5 μL) at a rate of 0.4 μL/minute. At the designated time points, mice were perfused transcardially with PBS and 4% paraformaldehyde (PFA), brains were removed and underwent overnight fixation in 4% PFA. After perfusion and fixation, tissues were processed into paraffin via sequential dehydration and perfusion with paraffin under vacuum. Brains were then embedded in paraffin blocks, cut into 6 μm sections and mounted on glass slides for immunofluorescence staining.

### Human brain tissue

Substantia nigra paraffin-embedded tissues from 5 idiopathic Lewy body disease brains and 7 healthy matched controls were examined. All procedures were done in accordance with local institutional review board guidelines of the Van Andel Institute Brain Bank, Van Andel Institute, Grand Rapids, MI, USA. Written informed consent for autopsy and analysis of tissue sample data was obtained either from patients themselves or their next of kin. Tissues were selected based on neuropathological diagnoses. Non-pathological cases were balanced by age, sex, and PMI.

### Immunofluorescence

Slides were de-paraffinized with 2 sequential 5-minute washes in xylenes, followed by 1-minute washes in a descending series of ethanol: 100%, 100%, 95%, 80%, 70%. Slides were then incubated in deionized water for one minute prior to transfer to the BioGenex EZ-Retriever System where they were incubated in antigen unmasking solution (Vector Laboratories; Cat# H-3300) and microwaved for 15 minutes at 95°C. Slides were allowed to cool for 20 minutes at room temperature and washed in running tap water for 10 minutes. Slides were washed 5 minutes in 0.1 M Tris (diluted from 0.5 M Tris made from Tris base and concentrated hydrochloric acid to pH 7.6), then blocked in 0.1 M Tris/2% fetal bovine serum (FBS) for 1 hour. Slides were incubated in primary antibody in 0.1 M Tris/2% FBS in a humidified chamber overnight at 4°C; pS129 α-synuclein (EP1536Y, Abcam ab51253, RRID: AB_869973, 1:5000); pS129 α-synuclein (81A), BioLegend 825701, RRID: AB_256489, 1:1000; Guinea Pig NeuN (Synaptic Systems 266 004, RRID: AB_2619988, 1:1000), pPAK(GII), Cell Signaling 3241, RRID: AB_2158623, 1:500). Primary antibody was rinsed off with 0.1 M tris for 5 minutes and incubated with secondary antibodies (Goat anti-Rabbit IgG 647, Thermo A-21244, RRID: AB_2535812, 1:1000); (Goat anti-Guinea Pig IgG 568, Thermo A-11075, RRID: AB_2534119, 1:1000) on slides in the dark for 3 hours at room temperature, rinsed and washed 0.1 M Tris for 10 minutes three times, then mounted with coverglass in ProLong gold with DAPI (Invitrogen, Cat#P36931). Fluorescent slides were imaged at 20x magnification on a Zeiss AxioScan 7 microscope or on the ImageXpress Confocal HT.ai High-Content Imaging System at 60x magnification.

### Mouse brain segmentation and registration

#### Segmentation

Stained slides were scanned on a Zeiss AxioScan 7 at 20x magnification and imported into QuPath v0.5.0 (RRID: SCR_018257)^86^ for analysis. A pixel classifier thresholding fluorescent intensity for the pS129 α-synuclein channel was applied to each section, picking up positive pathology signal for quantification. Signal Intensity was optimized by adjusting the min/max display settings. A threshold with similar settings was applied across cohorts. Individual cells were identified using the Cell Detection feature in QuPath that detects cells based on DAPI. Cell detection parameters such as background radius, sigma, and threshold were adjusted to optimize cell detection across brain sections. An object classifier identifying cells positive for pS129 α-synuclein was applied to sections. QuPath was trained on a subset of annotations to ensure proper classification of positive cells based on signal intensity. Once sufficiently accurate, the classifier was loaded onto the entire brain image to classify all detected cells. The subcellular spot detection feature was used to identify pS129 α-synuclein signal within positive pS129 α-synuclein cells for the inclusion area measure. The same fluorescent intensity level set for the pixel thresholder was applied for subcellular spot detections.

#### Mouse Coronal Section Brain Registration

Images were registered to the Allen Brain Atlas CCFv3 using a modified version of the QUINT workflow (RRID: SCR_023856). An RGB image of each section was exported from QuPath as a PNG, down sampled by a factor of 12, to use for spatial registration in QuickNII (RRID:SCR_016854). Segmentation was created by exporting a color-coded image of classified pixels or cells on a white background for use as the segmentation input in Nutil (RRID:SCR_017183). Brain images were uploaded to the web version of DeepSlice (RRID: SCR_023854) and the deep neural network was used to automatically align sections to regions of the atlas. Preliminary alignment was exported from DeepSlice as an XML that was then uploaded to QuickNII (RRID: SCR_016854) for further refinement. Following the spatial registration of the mouse brain sections to the Allen Mouse Brain Atlas CCFv3 in QuickNII, a JSON file was saved for use in VisuAlign (RRID:SCR_017978). Brain sections were imported into VisuAlign to fine tune the registrations to match regions of interest. Anchor points were generated in the atlas overlay and moved to the corresponding location on the brain section via non-linear transformations. Markers were placed around the contour of the brain section first with markers refining the inner structures applied second. Final alignments were exported as FLAT and PNG files for use in Nutil.

Nutil was used for the quantification and spatial analysis of the identified cell types in specific regions of the mouse brain. Each segmentation (pixels and cells) was run through Nutil by uploading the input files generated from the masks pulled out in QuPath, a JSON anchoring file from QuickNII, and a folder with the FLAT and PNG files from VisuAlign containing the transformed registration. Individual classes were identified for quantification via their HTML color code assigned in QuPath. Nutil generated object area, region area, area occupied, and object counts from each individual classification within each region of the Allen Mouse Brain Atlas using the registration from QuickNII and VisuAlign. The outputs are used in modeling and in N2U to generate anatomical heatmaps.

#### 3D Mouse Brain Registration and Segmentation

The α-synuclein channel from the SPIM data set (tiff image stack) was imported into Fiji/ImageJ v1.54f^87^ for preprocessing (manual threshold and masking with CLIJ2) and saved as a multipage tiff prior to registration to the ABA CCFv3 using BrainGlobe’s BrainReg software^88^. The following key parameters were used for registration: voxel size: 4.0 z, 1.8 xy; orientation: sal; geometry: full brain; atlas: 10 μm; save original orientation: True. The exported brain atlas label values were remapped to 16-bit space for easier downstream analysis using a custom python script and then up sampled in Fiji to half the size of the original dataset in xyz. The hemisphere mask generated by BrainReg was transposed in yz with CLIJ2^89^ and used to uniquely label all brain regions. The original SPIM dataset in the pS129 α-synuclein channel was relabeled using a 16-bit atlas, and the transposed hemisphere images were all imported into Imaris (Oxford Instruments) as a single ims file. The relabeled 16-bit atlas was additionally imported as a label/segmentation image for automatic conversion into Surface objects. The pS129 α-synuclein signal was then segmented with the Surfaces module using automatic filtering parameters and a surface grain size of 0.5 μm. Overlap between brain region surfaces and segmented α-synuclein surfaces was calculated with Imaris’ Object-Object statistics option. Quantitative information was exported for further analysis.

### Anatomical Analysis Heatmaps

#### Nutil-to-Usable (N2U)

Nutil-to-Usable is an R-based shiny app developed by bioinformaticians at Van Andel Institute designed to aid the plotting of regional data to anatomical heatmaps based on the Allen Brain Atlas (RRID:SCR_024753). Outputs from Nutil were uploaded with data from the left and right hemispheres submitted separately. An annotation file is generated allowing for labelling of metadata for each section’s associated Nutil file (mouse id, sex, treatment, post-injection interval, etc.). Checkpoint files compiling the raw Nutil files and metadata were downloaded to facilitate faster upload times when working downstream in the app. Settings were then defined to determine the level of summary and variable of interest sought to average. ‘Load’, defined as the area of pathology/area of the region, was set as variable of interest when plotting for total and cell body inclusion area occupied. To plot neurite area occupied, checkpoint files from total and inclusion were combined and uploaded with the app variable of interest set to ‘Neurite Load’. The app runs calculations to subtract cell body inclusion area from total area for each region and run relevant calculations to divide by region area resulting in neurite area occupied. Level of resolution for the atlas is set to ‘daughter’ (more specific, individual layers and subregions shown), or ‘parent’ (more general, layers compiled into single region). Grid Heatmaps and plotting matrices are developed in this step.

#### Mouse Brain Heatmaps (MBH)

Plotting matrices were uploaded to MBH to generate anatomical heatmaps. MBH plots the compiled data for the parameter of interest represented in the plotting matrix set by N2U. Applicable coronal section figures were listed in the app by number based on the ordering of Allen Brain Atlas sections. Regional Resolution for the plots was set to either daughter (by layer) or parent (compiling layers). Plots were downloaded as svgs.

#### Statistical Analysis

For analyses assessing differences in percent area occupied between timepoints, data were stratified based on brain hemisphere (ipsilateral/contralateral), brain region, and MPI. Regions with zero variance across all timepoints were filtered out. To maintain flexibility and avoid reliance on strong parametric assumptions, robust linear regressions via the MASS package, with the ranked percent area occupied as the outcome and MPI as the explanatory variable, were used (https://cran.r-project.org/web/packages/MASS/index.html, RRID: SCR_019125). All models were adjusted for sex and daughter regions when the number of daughter regions was 2 or greater. Effect sizes, including confidence intervals were estimated using the R package emmeans (R package version 1.8.3, URL: https://CRAN.R-project.org/package=emmeans, RRID: SCR_018734). Significance was determined using second-generation P values based on a null interval of ± 5% difference with 95% confidence intervals. Only second-generation P values equal to 0 were considered significant.

### GeoMx spatial transcriptomics

#### Tissue preparation

Spatial transcriptomics was performed using the nanoString GeoMx^®^ Digital Spatial Profiler. Sections were cut at 6 μm thickness and mounted on plus-charged slides (Epredia Colormark Plus CM-4951WPLUS-001). Slides were baked at 60°C for 1 hour and stored at 4°C in a vacuum sealed container containing desiccant for up to two weeks. All subsequent steps were performed using RNase-free conditions and DEPC treated water. Slides were de-paraffinized with 3 sequential 5-minute washes in xylenes, followed by 2 washes in 100% ethanol for 5 minutes, 1 wash in 95% ethanol, and 1 wash in 1x PBS. Target retrieval was performed in target retrieval reagent (10x Invitrogen 00-4956-58 EDTA pH 9.0) diluted to 1x in the BioGenex EZ-Retriever System for 10 minutes at 95°C. Slides were then washed with 1x PBS for 5 minutes. Slides were then incubated in 0.1μg/mL proteinase K (Invitrogen 25530–049) for 10 minutes at 37°C and washed in 1x PBS for 5 minutes at room temperature. Slides were post fixed for 5 minutes in 10% neutral buffered formalin followed by two washes in NBF stop buffer (24.5g Tris base and 15g Glycine in 2L DEPC water) for 5 minutes each and one wash in 1x PBS for 5 minutes. Slides were then incubated with hybridization probes (nanoString Cat# 121401103) diluted in Buffer R (provided in the GeoMx RNA Slide Prep FFPE- PCLN kit, catalog # 121300313) in a hybridization oven at 37°C for 16–20 hours.

Following probe incubation, slides were washed with stringent washes (equal parts formamide and 4x SSC buffer) at 37°C twice for 25 minutes each. Then slides were washed twice in 2x SSC buffer. Slides were incubated in 200 μL buffer W (provided in the GeoMx RNA Slide Prep FFPE- PCLN kit, catalog # 121300313) for 30 minutes and incubated in morphology markers (GFAP-488, ThermoFisher Scientific 53-9892-82, RRID:AB_10598515, 1:400; HuC/HuD (16A11) ThermoFisher Scientific A21271, RRID:AB_221448,1:500; NeuN, Millipore ABN78, RRID:AB_10807945, 1:1000) at 4°C overnight. The first four mice were characterized with NeuN only as the morphology marker. NeuN did not show efficient staining of several subcortical regions, so HuC/HuD were optimized for these regions and the last two mice included NeuN and HuC/HuD antibodies to enable segmentation of subcortical neuron types. Slides were washed 4 times in 2x SSC buffer for 3 minutes each wash. Slides were then incubated with secondary antibodies (GαRb 647, ThermoFisher Scientific A21244, RRID:AB_2535812, 1:1000; GαIgG2b 647 Thermo A21242, RRID:AB_2535811, 1:1000) and nuclei marker Syto83 (Thermo Scientific S11364, 1:1000) in Buffer W for 1 hour at room temperature in a humidified chamber. Slides were washed 4 times in 2x SSC buffer for 3 minutes each and placed in the nanoString GeoMx^®^ DSP instrument.

Syto83 immunofluorescence was utilized for autofocus of GeoMx imaging. Immunofluorescence for GFAP was used in identification of morphological markers to aid in fitting to the Allen Brain Institute’s mouse brain atlas. NeuN and HuC/HuD were used for segmentation. Prior to region-of-interest (ROI) generation, the slide image was exported, and individual brains were registered to the Allen Brain Atlas CCFv3^90^ using the mouse brain registration protocol. Images of registered brains were imported onto the DSP instrument and fit exactly to the slide image to enable accurate anatomical selection of ROIs. ROIs were generated using the polygon or circle tool which aligned with the correct brain region. Each ROI was segmented into one area-of-illumination (AOI) to detect neurons within the designated ROIs. Probe identities in each segment were captured via UV illumination and moved to a 96-well plate.

#### NGS library prep and sequencing

Library preps and sequencing were performed by the Van Andel Genomics Core. Illumina Novaseq 6000 was used for sequencing with a read length of 27 for both reads with reverse sequence orientation in the readout group plate information. Plates were dried down and rehydrated in 10 μL nuclease-free water, mixed and incubated at room temperature for 10 minutes. PCR was performed on samples as described in the nanoString GeoMx^®^ DSP Readout User Manual using 2 μL PCR master mix, 4 μL primer from the correct wells, and 4 μL resuspended DSP aspirate. KAPA beads (KAPA Pure beads, Roche Cat# 07983298001) were warmed to room temperature for 30 minutes. Libraries were pooled and KAPA beads were added to each pool at a 1.2X ratio to the final pool volume. Two KAPA bead clean ups were performed and pooled libraries were eluted in 24 μL elution buffer. Negative and positive control pools were eluted into 10 μL elution buffer. Quantity of the pools were assessed using the QuantiFluor^®^ dsDNA System (Promega Corp.). Pools were diluted to 5 ng/μL and quality and size are assessed using Agilent DNA High Sensitivity chip (Agilent Technologies, Inc.) on the Bioanalyzer. Sequencing was performed at 100 reads/μm^2^. Paired end 50 base paired sequencing. Base calling was done by Illumina RTA3 and output was demultiplexed and converted to fastq format with bcl2fastq v1.9.0. Fastq files were then converted to Digital count conversion files (DCC) using GeoMx NGS Pipeline v2.3.3.10. Sequencing reads were trimmed to 27 bp to reflect GeoMx probe length.

#### Quality Control and Data Analysis

In the PANGEA experiment, 717 segments were captured in total from 7 mice (3 male and 4 female; age=3 months), and 16 segments failed quality control (QC) analysis and were removed. One segment was removed for trimmed reads < 80%, stitched reads < 80%, and aligned reads < 75%. 14 additional segments were removed for sequence saturation below 50%. One additional segment was removed for < 3% of genes being detected above the limit of quantification (LOQ). Of the 20,175 gene targets contained in the GeoMx Mouse Whole Transcriptome Atlas, 19781 were included in the downstream analyses following QC analysis. 1 gene target was removed as a global outlier (Grubbs test, P<0.01), and 10 were removed as local outliers (Grubbs test, P<0.01). Of the remaining gene targets, 19,781 gene targets were detected above the LOQ in at least 1% of segments, and therefore were selected for further analysis. An additional 6 gene targets were identified as interesting *a priori* and were retained in the study for additional analysis, regardless of QC performance. Sample size was determined based on pilot data. No statistical method was used to determine sample size. Experiments did not involve multiple experimental conditions.

The PANGEA app was developed as a server-free web application designed to visualize and analyze brain region gene expression data. Developed using the Lume Core platform by Lume VR Limited (https://www.lumevr.com, Oxford, UK; contact: alexandre.kitching@lumevr.com), PANGEA offers a novel, ultra-low-maintenance solution with no ongoing operational costs, ensuring long-term accessibility and sustainability. This server-free architecture means that all data uploaded by end-users remains solely on their local machines, never shared or stored externally, which allows for secure analysis of proprietary or sensitive data. The platform hosts spatial gene expression data from mouse neuroanatomical regions, mapped onto the ABA CCFv3, enabling researchers to explore transcriptomic profiles in anatomically defined brain regions with ease and security.

In the mouse GeoMx experiment data used for Fig. 6H and 8G, and the human GeoMx experiment used for Fig. 8G, post-QC data was downloaded from the publicly available data previously published by our group, and utilized without additional alterations (https://doi.org/10.5281/zenodo.10729767)^32^. This data was originally produced using similar QC analyses as described above. For detailed QC parameters of this data see https://github.com/Goralsth/Spatial-transcriptomics-reveals-molecular-dysfunction-associated-with-cortical-Lewy-pathology (DOI: 10.5281/zenodo.10732492).

### Primary Neuron Culture

Primary neuron cultures were prepared from embryonic day 18 (E18) CD1 (strain 022; RRID: IMSR_CRL:022) mice. Brains were gently removed from the embryos and placed into a petri dish filled with ice-cold, sterile Hibernate Medium (Cat#. A1247601, Gibco) The hemispheres were gently separated, and the meninges, thalamus, striatum, brainstem, and hippocampus were removed. Hippocampi were isolated, pooled and digested in papain solution (20 U/mL Cat# LS003126, Worthington) and then treated with DNase I (Cat# LS006563, Worthington) to remove residual DNA. The tissue was then washed with pre-warmed Neurobasal media (Cat# 21103049, Gibco), mechanically dissociated, and strained through a 40 μm cell strainer. The cell suspension was pelleted at 1000 × *g* for 5 minutes, resuspended in 2 mL of neuron media (Neurobasal media containing 1% B27, 2 mM GlutaMAX, and penicillin-streptomycin), and gently mixed. The dissociated neurons were seeded on poly-D-lysine (Cat# P0899, Sigma) coated 96-well culture plates (Cat# 655090, Greiner) at 17,000 cells/well. Cells were maintained at 37°C at 5% CO_2_.

### Kinase Inhibitor Screen

All 12 compounds for the kinase screen and 7 PAK inhibitors were procured from MedChemExpress (listed in the Key Resources Table). Each compound was reconstituted in dimethyl sulfoxide (DMSO-D2438, Sigma-Aldrich) to create a stock solution at its maximum solubility. The three treatment doses were determined based on the compounds’ IC50 values for the target kinase. The stock solutions were further diluted with DMSO and aliquoted in a 96-well plate to the desired concentration for the treatment ensuring the final concentration of DMSO remains <0.1% at all tested dosages. On the day of treatment, the diluted compounds were mixed with neuron media and added to the primary neurons cultured in 96-well plates.

### Immunocytochemistry

Neurons were fixed with prewarmed 4% paraformaldehyde/4% sucrose in phosphate buffer saline (PBS) for 15 mins, followed by 5 washes with PBS. Cells were then permeabilized with 0.3% Triton X-100 in 3% bovine serum albumin (BSA) for 15 mins followed by a 3X wash with PBS. Cells were blocked with 3% BSA for 1 hour before incubation with primary antibodies (MAP2, Abcam AB5543, RRID: AB_571049, 1:5000; NeuN, Millipore MAB377, RRID: AB_2298767, 1:1500; pS129 α-synuclein (81A), BioLegend 825701, RRID: AB_256489, 1:2000; pPAK(GII), Cell Signaling 3241, RRID: AB_2158623, 1:500) at room temperature for 2 hours. Cells were washed 5X with PBS and were then incubated with fluorescent secondary antibodies (Goat anti-chicken-488, Thermo Scientific-A11039, RRID: AB_2534096; Goat anti mouse IgG2a-546, Thermo Scientific-A21133, RRID: AB_2535772; Goat anti mouse IgG1–647, Thermo Scientific- A21240, RRID: AB_2535809) for 1 hour at room temperature in dark, followed by 5 washes with PBS. All fluorescent secondary antibodies were used at a dilution of 1:500. Cells were then stained, incubated with DAPI, 1:10,000 in PBS. The plates were sealed and stored at 4°C until imaging.

### Imaging and image analysis

96-well plates of stained primary neurons from the kinase screen were imaged on ImageXpress Confocal HT.ai High-Content Imaging System at 10X magnification. 9 fields-of-view per well were imaged and imported into MetaXpress Analysis software (V3.7). MAP2/pS129 α-synuclein positive signal was segmented using ‘Find Blobs’ tool with minimum width of 1 μm and maximum width of 30 μm and 50 μm for MAP2 and pS129 α-synuclein respectively. NeuN positive nuclei were segmented with minimum width of 9 μm and maximum width of 45 μm. The threshold for each channel was kept the same for the entire plate and adjusted to only quantify true positive signal to background noise. Total area covered for MAP2 and pS129 α-synuclein and number of NeuN positive nuclei was quantified for each image. 96-well plates from the suite of PAK inhibitor screen were imaged on Zeiss Cell Discoverer 7 at 20X magnification with 0.5X digital zoom. 10 ROI per well were imaged. Images were analyzed on Zeiss Blue Analysis software (V3.7). The ‘Automatic segmentation’ tool was used to segment each signal. For MAP2 and pS129 α-synuclein, ‘Minimum Object Size’ was chosen 1 and threshold was set for each plate manually to segment most of the MAP2 and pS129 α-synuclein signal. For NeuN and DAPI; Minimum Object Size’ was chosen to be 200 and minimum and maximum thresholds were set manually for each plate to segment the NeuN and DAPI signal. Background thresholds were adjusted for true positive to background signal. NeuN and DAPI positive cells were counted and the area of MAP2 and pS129 α-synuclein were measured for each image. Data from all images/well was pooled, averaged, normalized to control wells and represented in terms of fold change.

### Statistical analysis

GraphPad Prism software version 10.2.2 (GraphPad Software Inc., La Jolla, CA, USA) was used for statistical analysis for primary neuron culture experiments.

### Computational modeling of α-synuclein pathology spread

#### Network spreading dynamics

Our model assumes that α-synuclein pathology proceeds bidirectionally in a linear fashion along the brain’s structural connectome. The structural connectome was constructed by estimating the projection strength between each pair of 100 μm-wide voxels within the Allen Institute CCFv3 whole-brain parcellation^91^. α-synuclein spread in both anterograde and retrograde directions was modeled according to the equation below:

$$\hat{y}(t)=b_{0}+b_{a}\;{\text{log}}_{\text{10}}(f(e^{-c_{a}l_{a}t}x_{0}))+b_{r}\;{\text{log}}_{\text{10}}(f(e^{-c_{r}l_{r}t}x_{0}))+\varepsilon (t)$$

where *b*_0_ is an intercept, *b*_*a*_ represents the importance of anterograde spread, *b*_*r*_ represents the importance of retrograde spread, *t* is time, and *ε* is an error term.

#### Network model validation

We used two established procedures to assess the performance of our predictive model^27,30,92^. First, to evaluate the model’s specificity to the experimental seed site, we randomly selected 500 alternate seed regions and redefined the vector x_0_ accordingly. We then refit the model for each alternate seed region to obtain a distribution of fits. For each time point, we obtained a nonparametric *p* value that reflects the proportion of times that the fit obtained from the actual seed site was stronger than that resulting from fitting the model to alternate seeds.

The linear diffusion model used here assumes that the spread of pathological α-synuclein proceeds bidirectionally. Model parameters, including diffusion rate constants and time-dependent weights for anterograde and retrograde spread, are fit using the available data from all mice at every time point. In order to statistically compare the out-of-sample performance of our bidirectional diffusion model to models based on anterograde spread alone, retrograde spread alone, or Euclidean distance, we randomly sampled the data to obtain *y*_*train*_*(t)* and *y*_*test*_*(t)* for each time point. Parameters were estimated using the model fitting process described in the preceding section on *y*_*train*_*(t)*, and the model’s performance was evaluated by its fit with *y*_*test*_*(t)*. This procedure was repeated 500 times for each model type to generate a distribution of model fits.

#### Regional vulnerability

In our linear diffusion model, *ε*(*t*) represents residual variance that is not explained by spread along the brain’s structural connectome. Positive values of *ε*(*t*) indicate that a region was occupied with higher levels of pathology than would be predicted by structural connectivity alone, and negative values of *ε*(*t*) indicate that a region accumulated lower levels of pathology than would be predicted by structural connectivity alone. To obtain a measure of regional vulnerability to pathology, we averaged the residual variance across the 1, 3, 6 and 9 MPI time points. Earlier time points (0.1, 0.2, 0.3, and 0.5 MPI) were excluded from the measure because model performance was weaker at those time points and patterns of residuals were dissimilar from later time points (Fig. S4D).

A scaled sigmoidal transformation was used to normalize levels of gene expression across the brain for each gene, such that all expression levels fall on a scale of 0 to 1. A rank inverse normalization transformation was then applied to both regional vulnerability and gene expression data. To identify genes that are associated with regional vulnerability, we computed Pearson’s correlations between regional vulnerability and gene expression for each gene in the PANGEA dataset. p-values were FDR corrected for multiple comparisons.

## Supplementary Material

Supplement 1

## Data Availability

The data that support the findings of this study together with the code used for data analysis are available here: https://github.com/Goralsth/Synuclein_Progression_Github. Primary code used to analyze data and generate linear diffusion models is available here: https://github.com/jkbrynildsen/aSyn_spread. Initial conversion of Nutil files to matrix output and grid heatmap generation: https://github.com/DaniellaDeWeerd/NutilToUsable (RRID:SCR_024753). Brain heatmaps and differential analysis of area occupied: https://github.com/vari-bbc/Mouse_Brain_Heatmap.

## HIGHLIGHTS

- Longitudinal α-synuclein pathology assessment in 1046 brain regions over 9 months
- Linear diffusion modeling derivation of network vulnerability to α-synuclein pathology
- PANGEA: assessment of over 19,000 genes in 302 brain regions
- Group II PAK inhibitor prevents α-synuclein pathology and neuron death

## References

[1] Kalia L. V. & Lang A. E. Parkinson’s disease. Lancet 386, 896–912, doi:10.1016/S0140-6736(14)61393-3 (2015). 25904081

[2] Bloem B. R., Okun M. S. & Klein C. Parkinson’s disease. Lancet 397, 2284–2303, doi:10.1016/S0140-6736(21)00218-X (2021). 33848468

[3] Coukos R. & Krainc D. Key genes and convergent pathogenic mechanisms in Parkinson disease. Nature reviews. Neuroscience 25, 393–413, doi:10.1038/s41583-024-00812-2 (2024). 38600347

[4] McGregor M. M. & Nelson A. B. Circuit Mechanisms of Parkinson’s Disease. Neuron 101, 1042–1056, doi:10.1016/j.neuron.2019.03.004 (2019). 30897356

[5] Borghammer P. Neuropathological evidence of body-first vs. brain-first Lewy body disease. Neurobiology of disease 161, 105557, doi:10.1016/j.nbd.2021.105557 (2021). 34763110

[6] Brettschneider J., Del Tredici K., Lee V. M. & Trojanowski J. Q. Spreading of pathology in neurodegenerative diseases: a focus on human studies. Nature reviews. Neuroscience 16, 109–120, doi:10.1038/nrn3887 (2015). 25588378 PMC4312418

[7] McFarthing K. Parkinson’s Disease Drug Therapies in the Clinical Trial Pipeline: 2024 Update. Journal of Parkinson’s disease 14, 899–912, doi:10.3233/JPD-240272 (2024). PMC11307066 39031388

[8] Maschio C. & Ni R. Amyloid and Tau Positron Emission Tomography Imaging in Alzheimer’s Disease and Other Tauopathies. Frontiers in aging neuroscience 14, 838034, doi:10.3389/fnagi.2022.838034 (2022). 35527737 PMC9074832

[9] Raj A., Kuceyeski A. & Weiner M. A network diffusion model of disease progression in dementia. Neuron 73, 1204–1215, doi:10.1016/j.neuron.2011.12.040 (2012). 22445347 PMC3623298

[10] Pandya S. Predictive model of spread of Parkinson’s pathology using network diffusion. NeuroImage 192, 178–194, doi:10.1016/j.neuroimage.2019.03.001 (2019). 30851444 PMC7180066

[11] Pandya S., Mezias C. & Raj A. Predictive Model of Spread of Progressive Supranuclear Palsy Using Directional Network Diffusion. Frontiers in neurology 8, 692, doi:10.3389/fneur.2017.00692 (2017). 29312121 PMC5742613

[12] Dagher A. & Zeighami Y. Testing the Protein Propagation Hypothesis of Parkinson Disease. Journal of experimental neuroscience 12, 1179069518786715, doi:10.1177/1179069518786715 (2018). PMC6043918 30013389

[13] Fornari S., Schafer A., Jucker M., Goriely A. & Kuhl E. Prion-like spreading of Alzheimer’s disease within the brain’s connectome. Journal of the Royal Society, Interface 16, 20190356, doi:10.1098/rsif.2019.0356 (2019). 31615329 PMC6833337

[14] Ossenkoppele R. Tau covariance patterns in Alzheimer’s disease patients match intrinsic connectivity networks in the healthy brain. NeuroImage. Clinical 23, 101848, doi:10.1016/j.nicl.2019.101848 (2019). 31077982 PMC6510968

[15] Zheng Y. Q. Local vulnerability and global connectivity jointly shape neurodegenerative disease propagation. PLoS biology 17, e3000495, doi:10.1371/journal.pbio.3000495 (2019). 31751329 PMC6894889

[16] Schafer A., Mormino E. C. & Kuhl E. Network Diffusion Modeling Explains Longitudinal Tau PET Data. Frontiers in neuroscience 14, 566876, doi:10.3389/fnins.2020.566876 (2020). 33424532 PMC7785976

[17] Vogel J. W. Spread of pathological tau proteins through communicating neurons in human Alzheimer’s disease. Nature communications 11, 2612, doi:10.1038/s41467-020-15701-2 (2020). PMC7251068 32457389

[18] Tremblay C. Brain atrophy progression in Parkinson’s disease is shaped by connectivity and local vulnerability. Brain Commun 3, fcab269, doi:10.1093/braincomms/fcab269 (2021). 34859216 PMC8633425

[19] Franzmeier N. Tau deposition patterns are associated with functional connectivity in primary tauopathies. Nature communications 13, 1362, doi:10.1038/s41467-022-28896-3 (2022). PMC8924216 35292638

[20] Frontzkowski L. Earlier Alzheimer’s disease onset is associated with tau pathology in brain hub regions and facilitated tau spreading. Nature communications 13, 4899, doi:10.1038/s41467-022-32592-7 (2022). PMC9392750 35987901

[21] Lee W. J. Regional Abeta-tau interactions promote onset and acceleration of Alzheimer’s disease tau spreading. Neuron 110, 1932–1943 e1935, doi:10.1016/j.neuron.2022.03.034 (2022). 35443153 PMC9233123

[22] Vogel J. W. Connectome-based modelling of neurodegenerative diseases: towards precision medicine and mechanistic insight. Nature reviews. Neuroscience 24, 620–639, doi:10.1038/s41583-023-00731-8 (2023). 37620599

[23] Ottoy J. Tau follows principal axes of functional and structural brain organization in Alzheimer’s disease. Nature communications 15, 5031, doi:10.1038/s41467-024-49300-2 (2024). PMC11169286 38866759

[24] Bidesi N. S. R., Vang Andersen I., Windhorst A. D., Shalgunov V. & Herth M. M. The role of neuroimaging in Parkinson’s disease. Journal of neurochemistry 159, 660–689, doi:10.1111/jnc.15516 (2021). 34532856 PMC9291628

[25] Luk K. C. Pathological alpha-synuclein transmission initiates Parkinson-like neurodegeneration in nontransgenic mice. Science 338, 949–953, doi:10.1126/science.1227157 (2012). 23161999 PMC3552321

[26] Guo J. L. Unique pathological tau conformers from Alzheimer’s brains transmit tau pathology in nontransgenic mice. The Journal of experimental medicine 213, 2635–2654, doi:10.1084/jem.20160833 (2016). 27810929 PMC5110027

[27] Cornblath E. J. Computational modeling of tau pathology spread reveals patterns of regional vulnerability and the impact of a genetic risk factor. Sci Adv 7, doi:10.1126/sciadv.abg6677 (2021). PMC8189700 34108219

[28] Henderson M. Tau pathology spreads between anatomically-connected regions of the brain and is modulated by a LRRK2 mutation. bioRxiv, 2020.2010.2013.337857, doi:10.1101/2020.10.13.337857 (2020).

[29] Henderson M. X. Glucocerebrosidase Activity Modulates Neuronal Susceptibility to Pathological alpha-Synuclein Insult. Neuron 105, 822–836 e827, doi:10.1016/j.neuron.2019.12.004 (2020). 31899072 PMC7060125

[30] Henderson M. X. Spread of alpha-synuclein pathology through the brain connectome is modulated by selective vulnerability and predicted by network analysis. Nat Neurosci 22, 1248–1257, doi:10.1038/s41593-019-0457-5 (2019). 31346295 PMC6662627

[31] Henderson M. X. The roles of connectivity and neuronal phenotype in determining the pattern of alpha-synuclein pathology in Parkinson’s disease. Neurobiology of disease, 105687, doi:10.1016/j.nbd.2022.105687 (2022). 35283326 PMC9610381

[32] Goralski T. M. Spatial transcriptomics reveals molecular dysfunction associated with cortical Lewy pathology. Nature communications 15, 2642, doi:10.1038/s41467-024-47027-8 (2024). PMC10966039 38531900

[33] Yates S. C. QUINT: Workflow for Quantification and Spatial Analysis of Features in Histological Images From Rodent Brain. Frontiers in neuroinformatics 13, 75, doi:10.3389/fninf.2019.00075 (2019). 31849633 PMC6901597

[34] Henderson M. X. Regional Mouse Brain Analysis (Modified QUINT). protocols.io, doi:10.17504/protocols.io.kqdg3xbkzg25/v1 (2023).

[35] Rey N. L. Spread of aggregates after olfactory bulb injection of alpha-synuclein fibrils is associated with early neuronal loss and is reduced long term. Acta neuropathologica 135, 65–83, doi:10.1007/s00401-017-1792-9 (2018). 29209768 PMC5756266

[36] Bassil F. Amyloid-Beta (Abeta) Plaques Promote Seeding and Spreading of Alpha-Synuclein and Tau in a Mouse Model of Lewy Body Disorders with Abeta Pathology. Neuron 105, 260–275 e266, doi:10.1016/j.neuron.2019.10.010 (2020). 31759806 PMC6981053

[37] Geibl F. F. alpha-Synuclein pathology disrupts mitochondrial function in dopaminergic and cholinergic neurons at-risk in Parkinson’s disease. Molecular neurodegeneration 19, 69, doi:10.1186/s13024-024-00756-2 (2024). 39379975 PMC11462807

[38] Henrich M. T. Determinants of seeding and spreading of alpha-synuclein pathology in the brain. Sci Adv 6, doi:10.1126/sciadv.abc2487 (2020). PMC7673735 33177086

[39] Lein E. S. Genome-wide atlas of gene expression in the adult mouse brain. Nature 445, 168–176, doi:10.1038/nature05453 (2007). 17151600

[40] Fulcher B. D., Murray J. D., Zerbi V. & Wang X. J. Multimodal gradients across mouse cortex. Proc Natl Acad Sci U S A 116, 4689–4695, doi:10.1073/pnas.1814144116 (2019). 30782826 PMC6410879

[41] Ortiz C. Molecular atlas of the adult mouse brain. Sci Adv 6, eabb3446, doi:10.1126/sciadv.abb3446 (2020). 32637622 PMC7319762

[42] Yao Z. A high-resolution transcriptomic and spatial atlas of cell types in the whole mouse brain. Nature 624, 317–332, doi:10.1038/s41586-023-06812-z (2023). 38092916 PMC10719114

[43] Geertsma H. M. A topographical atlas of alpha-synuclein dosage and cell type-specific expression in adult mouse brain and peripheral organs. NPJ Parkinson’s disease 10, 65, doi:10.1038/s41531-024-00672-8 (2024). PMC10951202 38504090

[44] Kilfeather P. Single-cell spatial transcriptomic and translatomic profiling of dopaminergic neurons in health, aging, and disease. Cell reports 43, 113784, doi:10.1016/j.celrep.2024.113784 (2024). 38386560

[45] Murray B. W. Small-molecule p21-activated kinase inhibitor PF-3758309 is a potent inhibitor of oncogenic signaling and tumor growth. Proc Natl Acad Sci U S A 107, 9446–9451, doi:10.1073/pnas.0911863107 (2010). 20439741 PMC2889050

[46] Won S. Y. p21-activated kinase 4 controls the aggregation of alpha-synuclein by reducing the monomeric and aggregated forms of alpha-synuclein: involvement of the E3 ubiquitin ligase NEDD4–1. Cell death & disease 13, 575, doi:10.1038/s41419-022-05030-1 (2022). 35773260 PMC9247077

[47] Qu J. PAK4 kinase is essential for embryonic viability and for proper neuronal development. Molecular and cellular biology 23, 7122–7133, doi:10.1128/MCB.23.20.7122-7133.2003 (2003). 14517283 PMC230313

[48] Pandey A. Cloning and characterization of PAK5, a novel member of mammalian p21-activated kinase-II subfamily that is predominantly expressed in brain. Oncogene 21, 3939–3948, doi:10.1038/sj.onc.1205478 (2002). 12032833

[49] Nekrasova T., Jobes M. L., Ting J. H., Wagner G. C. & Minden A. Targeted disruption of the Pak5 and Pak6 genes in mice leads to deficits in learning and locomotion. Developmental biology 322, 95–108, doi:10.1016/j.ydbio.2008.07.006 (2008). 18675265

[50] Karpowicz R. J. Jr. Selective imaging of internalized proteopathic alpha-synuclein seeds in primary neurons reveals mechanistic insight into transmission of synucleinopathies. J Biol Chem 292, 13482–13497, doi:10.1074/jbc.M117.780296 (2017). 28611062 PMC5555207

[51] Kaur R. Activation of p21-activated kinase 6 by MAP kinase kinase 6 and p38 MAP kinase. J Biol Chem 280, 3323–3330, doi:10.1074/jbc.M406701200 (2005). 15550393

[52] Dadgar-Kiani E., Bieri G., Melki R., Gitler A. D. & Lee J. H. Mesoscale connections and gene expression empower whole-brain modeling of alpha-synuclein spread, aggregation, and decay dynamics. Cell reports 41, 111631, doi:10.1016/j.celrep.2022.111631 (2022). 36351406 PMC10840492

[53] Langlieb J. The molecular cytoarchitecture of the adult mouse brain. Nature 624, 333–342, doi:10.1038/s41586-023-06818-7 (2023). 38092915 PMC10719111

[54] Liu H. Single-cell DNA methylome and 3D multi-omic atlas of the adult mouse brain. Nature 624, 366–377, doi:10.1038/s41586-023-06805-y (2023). 38092913 PMC10719113

[55] Zhang M. Molecularly defined and spatially resolved cell atlas of the whole mouse brain. Nature 624, 343–354, doi:10.1038/s41586-023-06808-9 (2023). 38092912 PMC10719103

[56] Cahill R. Unsupervised pattern identification in spatial gene expression atlas reveals mouse brain regions beyond established ontology. Proc Natl Acad Sci U S A 121, e2319804121, doi:10.1073/pnas.2319804121 (2024). 39226356 PMC11406299

[57] Ali M. Z. & Dholaniya P. S. Oxidative phosphorylation mediated pathogenesis of Parkinson’s disease and its implication via Akt signaling. Neurochemistry international 157, 105344, doi:10.1016/j.neuint.2022.105344 (2022). 35483538

[58] Shoffner J. M., Watts R. L., Juncos J. L., Torroni A. & Wallace D. C. Mitochondrial oxidative phosphorylation defects in Parkinson’s disease. Ann Neurol 30, 332–339, doi:10.1002/ana.410300304 (1991). 1952821

[59] Ma S. X. Complement and Coagulation Cascades are Potentially Involved in Dopaminergic Neurodegeneration in alpha-Synuclein-Based Mouse Models of Parkinson’s Disease. Journal of proteome research 20, 3428–3443, doi:10.1021/acs.jproteome.0c01002 (2021). 34061533 PMC8628316

[60] Patterson J. R. Transcriptomic profiling of early synucleinopathy in rats induced with preformed fibrils. NPJ Parkinson’s disease 10, 7, doi:10.1038/s41531-023-00620-y (2024). PMC10764951 38172128

[61] Liddelow S. A. Neurotoxic reactive astrocytes are induced by activated microglia. Nature 541, 481–487, doi:10.1038/nature21029 (2017). 28099414 PMC5404890

[62] Loeffler D. A., Camp D. M. & Conant S. B. Complement activation in the Parkinson’s disease substantia nigra: an immunocytochemical study. Journal of neuroinflammation 3, 29, doi:10.1186/1742-2094-3-29 (2006). 17052351 PMC1626447

[63] Yamada T., McGeer P. L. & McGeer E. G. Lewy bodies in Parkinson’s disease are recognized by antibodies to complement proteins. Acta neuropathologica 84, 100–104, doi:10.1007/BF00427222 (1992). 1502878

[64] Jang Y. Mass Spectrometry-Based Proteomics Analysis of Human Substantia Nigra From Parkinson’s Disease Patients Identifies Multiple Pathways Potentially Involved in the Disease. Molecular & cellular proteomics : MCP 22, 100452, doi:10.1016/j.mcpro.2022.100452 (2023). 36423813 PMC9792365

[65] Chi X. Astrocyte-neuron communication through the complement C3-C3aR pathway in Parkinson’s disease. Brain, behavior, and immunity, doi:10.1016/j.bbi.2024.09.022 (2024). 39288893

[66] Vazquez-Velez G. E. Doublecortin-like Kinase 1 Regulates alpha-Synuclein Levels and Toxicity. J Neurosci 40, 459–477, doi:10.1523/JNEUROSCI.1076-19.2019 (2020). 31748376 PMC6948939

[67] Nagpal L., Kornberg M. D. & Snyder S. H. Inositol hexakisphosphate kinase-2 non-catalytically regulates mitophagy by attenuating PINK1 signaling. Proc Natl Acad Sci U S A 119, e2121946119, doi:10.1073/pnas.2121946119 (2022). 35353626 PMC9169102

[68] Di Leva F. Increased Levels of the Parkinson’s Disease-Associated Gene ITPKB Correlate with Higher Expression Levels of alpha-Synuclein, Independent of Mutation Status. International journal of molecular sciences 24, doi:10.3390/ijms24031984 (2023). PMC9916293 36768321

[69] Apicco D. J. The Parkinson’s disease-associated gene ITPKB protects against alpha-synuclein aggregation by regulating ER-to-mitochondria calcium release. Proc Natl Acad Sci U S A 118, doi:10.1073/pnas.2006476118 (2021). PMC7817155 33443159

[70] Arias-Romero L. E. & Chernoff J. A tale of two Paks. Biol Cell 100, 97–108, doi:10.1042/BC20070109 (2008). 18199048

[71] Radu M., Semenova G., Kosoff R. & Chernoff J. PAK signalling during the development and progression of cancer. Nat Rev Cancer 14, 13–25, doi:10.1038/nrc3645 (2014). 24505617 PMC4115244

[72] Kumar R., Sanawar R., Li X. & Li F. Structure, biochemistry, and biology of PAK kinases. Gene 605, 20–31, doi:10.1016/j.gene.2016.12.014 (2017). 28007610 PMC5250584

[73] Giusto E. Prospective Role of PAK6 and 14-3-3gamma as Biomarkers for Parkinson’s Disease. Journal of Parkinson’s disease 14, 495–506, doi:10.3233/JPD-230402 (2024). PMC11091598 38640169

[74] Strochlic T. I. Identification of neuronal substrates implicates Pak5 in synaptic vesicle trafficking. Proc Natl Acad Sci U S A 109, 4116–4121, doi:10.1073/pnas.1116560109 (2012). 22371566 PMC3306725

[75] Gao J. Substrate and inhibitor specificity of the type II p21-activated kinase, PAK6. PloS one 8, e77818, doi:10.1371/journal.pone.0077818 (2013). 24204982 PMC3810134

[76] Perez-Otano I. Endocytosis and synaptic removal of NR3A-containing NMDA receptors by PACSIN1/syndapin1. Nat Neurosci 9, 611–621, doi:10.1038/nn1680 (2006). 16617342 PMC1892311

[77] Zhang K., Wang Y., Fan T., Zeng C. & Sun Z. S. The p21-activated kinases in neural cytoskeletal remodeling and related neurological disorders. Protein Cell 13, 6–25, doi:10.1007/s13238-020-00812-9 (2022). 33306168 PMC8776968

[78] Mahfouz A. Genome-wide coexpression of steroid receptors in the mouse brain: Identifying signaling pathways and functionally coordinated regions. Proc Natl Acad Sci U S A 113, 2738–2743, doi:10.1073/pnas.1520376113 (2016). 26811448 PMC4791033

[79] Li X. & Minden A. Targeted disruption of the gene for the PAK5 kinase in mice. Molecular and cellular biology 23, 7134–7142, doi:10.1128/MCB.23.20.7134-7142.2003 (2003). 14517284 PMC230317

[80] Civiero L. Leucine-rich repeat kinase 2 interacts with p21-activated kinase 6 to control neurite complexity in mammalian brain. Journal of neurochemistry 135, 1242–1256, doi:10.1111/jnc.13369 (2015). 26375402 PMC4715492

[81] Iannotta L. PAK6 rescues pathogenic LRRK2-mediated ciliogenesis and centrosomal cohesion defects in a mutation-specific manner. Cell death & disease 15, 752, doi:10.1038/s41419-024-07124-4 (2024). 39419978 PMC11487180

[82] Zhao L. Role of p21-activated kinase pathway defects in the cognitive deficits of Alzheimer disease. Nat Neurosci 9, 234–242, doi:10.1038/nn1630 (2006). 16415866

[83] Volpicelli-Daley L. A., Luk K. C. & Lee V. M. Addition of exogenous alpha-synuclein preformed fibrils to primary neuronal cultures to seed recruitment of endogenous alpha-synuclein to Lewy body and Lewy neurite-like aggregates. Nature protocols 9, 2135–2146, doi:10.1038/nprot.2014.143 (2014). 25122523 PMC4372899

[84] Luk K. C. Exogenous alpha-synuclein fibrils seed the formation of Lewy body-like intracellular inclusions in cultured cells. Proc Natl Acad Sci U S A 106, 20051–20056, doi:10.1073/pnas.0908005106 (2009). 19892735 PMC2785290

[85] Volpicelli-Daley L. A. Exogenous alpha-synuclein fibrils induce Lewy body pathology leading to synaptic dysfunction and neuron death. Neuron 72, 57–71, doi:10.1016/j.neuron.2011.08.033 (2011). 21982369 PMC3204802

[86] Bankhead P. QuPath: Open source software for digital pathology image analysis. Scientific reports 7, 16878, doi:10.1038/s41598-017-17204-5 (2017). 29203879 PMC5715110

[87] Schindelin J. Fiji: an open-source platform for biological-image analysis. Nature methods 9, 676–682, doi:10.1038/nmeth.2019 (2012). 22743772 PMC3855844

[88] Tyson A. L. Accurate determination of marker location within whole-brain microscopy images. Scientific reports 12, 867, doi:10.1038/s41598-021-04676-9 (2022). 35042882 PMC8766598

[89] Haase R. CLIJ: GPU-accelerated image processing for everyone. Nature methods 17, 5–6, doi:10.1038/s41592-019-0650-1 (2020). 31740823

[90] Wang Q. The Allen Mouse Brain Common Coordinate Framework: A 3D Reference Atlas. Cell 181, 936–953 e920, doi:10.1016/j.cell.2020.04.007 (2020). 32386544 PMC8152789

[91] Knox J. E. High-resolution data-driven model of the mouse connectome. Netw Neurosci 3, 217–236, doi:10.1162/netn_a_00066 (2019). 30793081 PMC6372022

[92] Lubben N. LRRK2 kinase inhibition reverses G2019S mutation-dependent effects on tau pathology progression. Translational neurodegeneration 13, 13, doi:10.1186/s40035-024-00403-2 (2024). 38438877 PMC10910783

