# Supplementary material for: Network analysis of α-synuclein pathology progression reveals p21-activated kinases as regulators of vulnerability
Source: bioRxiv. 2024 Oct 22:2024.10.22.619411. Preprint. [Version 1] doi: 10.1101/2024.10.22.619411 (PMC11526907; doi:10.1101/2024.10.22.619411)
Supplement: Supplement 1 [file media-1.pdf]

Supplemental Figures for:

Network analysis of α-synuclein pathology progression reveals p21-activated kinases as regulators of vulnerability

Naman Vatsa[1,2#], Julia K. Brynildsen[2,3#], Thomas M. Goralski[1,2#], Kevin Kurgat[1,2#], Lindsay Meyerdirk[1,2], Libby Breton[1,2], Daniella DeWeerd[1,2], Laura Brasseur[1,2], Lisa Turner[10], Katelyn Becker[10], Kristin L. Gallik[10], Dani S. Bassett[2-9*], Michael X. Henderson[1,2*]

[1]Department of Neurodegenerative Science, Van Andel Institute, Grand Rapids, MI, USA
[2]Aligning Science Across Parkinson's (ASAP) Collaborative Research Network, Chevy Chase, MD, USA
[3]Department of Bioengineering, University of Pennsylvania, Philadelphia, PA, USA
[4]Department of Electrical & Systems Engineering, University of Pennsylvania, Philadelphia, PA, USA
[5]Department of Physics & Astronomy, University of Pennsylvania, Philadelphia, PA, USA
[6]Department of Neurology, University of Pennsylvania, Philadelphia, PA, USA
[7]Department of Psychiatry, University of Pennsylvania, Philadelphia, PA, USA
[8]Santa Fe Institute, Santa Fe, NM, USA
[9]Montreal Neurological Institute, McGill University, Montreal, Quebec, Canada
[10]Van Andel Institute, Grand Rapids, MI, USA

Figure S1

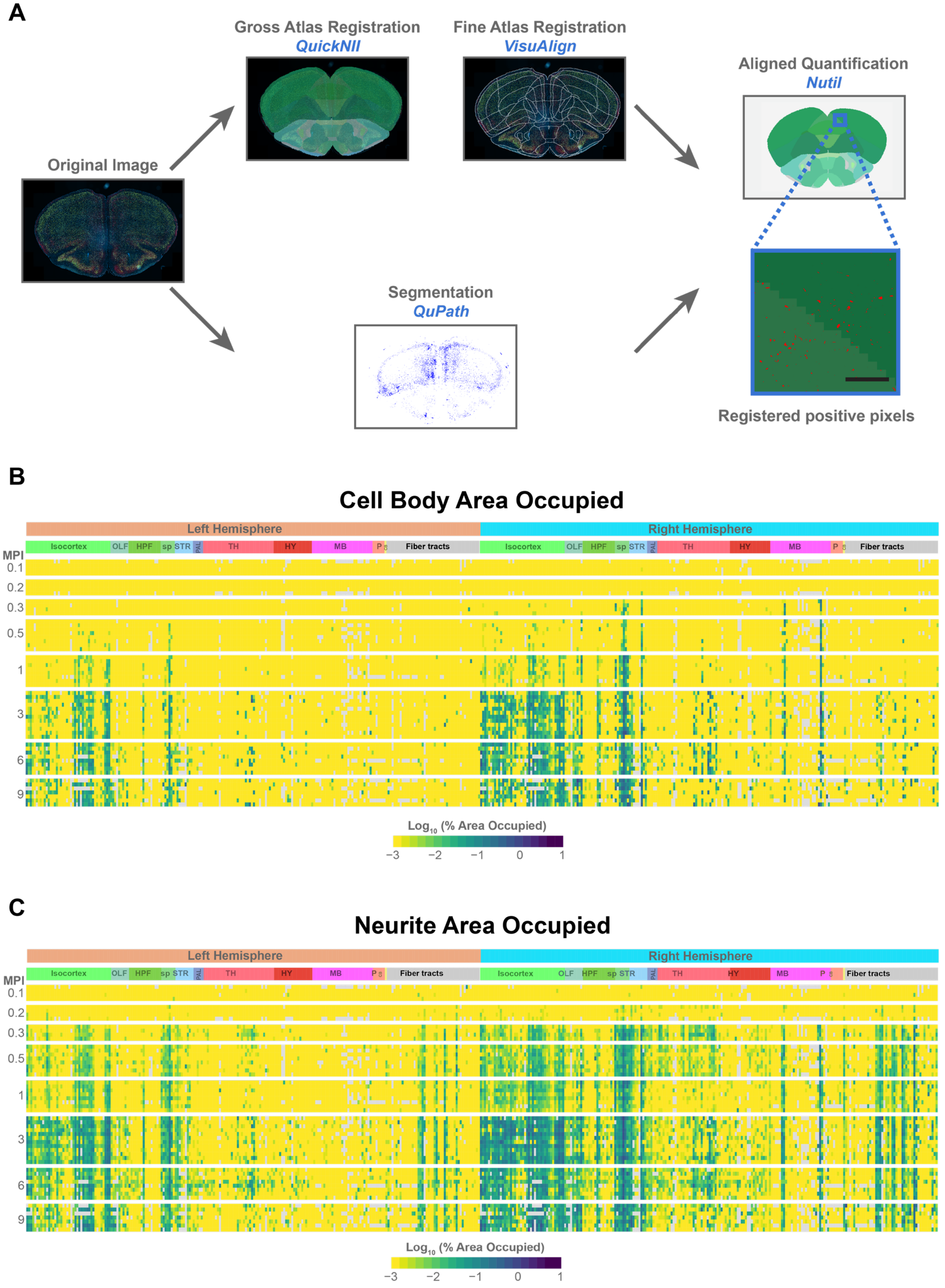
A
Gross Atlas Registration
QuickNII
Fine Atlas Registration
VisuAlign
Aligned Quantification
Nutil
Original Image
Segmentation
QuPath
Registered positive pixels
B
Cell Body Area Occupied
Left Hemisphere
Right Hemisphere
Isocortex
OLF
HPF
sp
STR
PAL
TH
HY
MB
P
Fiber tracts
MPI
0.1
0.2
0.3
0.5
1
3
6
9
Log10 (% Area Occupied)
−3
−2
−1
0
1
C
Neurite Area Occupied
Left Hemisphere
Right Hemisphere
Isocortex
OLF
HPF
sp
STR
PAL
TH
HY
MB
P
Fiber tracts
MPI
0.1
0.2
0.3
0.5
1
3
6
9
Log10 (% Area Occupied)
−3
−2
−1
0
1

Figure S1. Registration and segmentation of cell body and neuritic pathology (A) Schematic of the modified QUINT workflow used to analyze brain sections. Following staining, brain sections were digitized. Those digital images were registered to the ABA CCFv3 with QuickNII and VisuAlign software. Segmentation for total, cell body, and neuritic pathology was performed in QuPath. Segmentation and registration were then integrated to generate pathology measures in each anatomical region. (B) Heatmap plot of cell body regional pathology measures from 0.1-9 MPI. Each row represents an individual mouse, and each column represents a brain region with major regional designations labeled at the top. (C) Heatmap plot of neuritic regional pathology measures from 0.1-9 MPI. Each row represents an individual mouse, and each column represents a brain region with major regional designations labeled at the top.

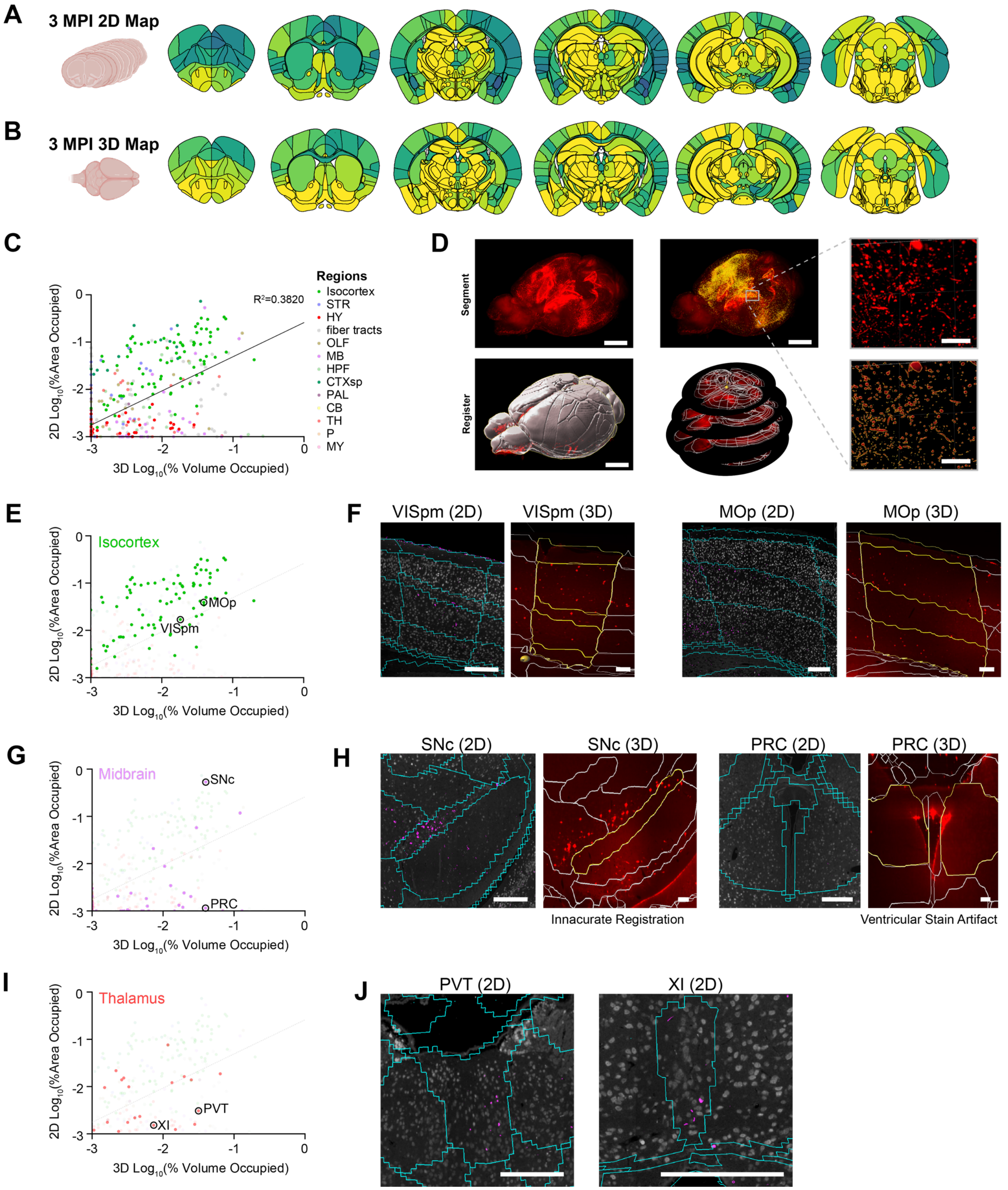

Figure S2. Comparison of 2D and 3D maps of α-synuclein pathology (A) Anatomical heatmap representing regional pathology area occupied for 3 MPI mice in our 2D analysis using the modified QUINT workflow. For the comparison to the 3D map, only cortical parent regions (and not laminar data) are shown. (B) Anatomical heatmap representing pathology volume occupied for 3 MPI mice in our 3D analysis. (C) Correlation plot showing individual

region pathology in the 2D and 3D analysis. Individual region data points are colored by major regional designations. Linear regression line of best fit and regression co-efficient are plotted. (D) Whole brain analysis methods. 3D reconstruction of mouse brain was rendered in IMARIS. Threshold generated pathology segmentation volume. Registration to the ABA CCFv3 via BrainGlobe registration. Scale bars = 2.5 mm for whole brain volume, 250 μm for zoomed segmentation. (E) Correlation plot highlighting regions within the isocortex. Cortical regions are generally well-correlated, with generally higher values in the 2D segmentation. (F) Selected visual (VISpm) and motor (MOp) cortical regions show similar amounts of pathology in both 2D and 3D analyses. Scale bars = 250 μm. (G) Correlation plot highlighting midbrain regions. (H) Selected regions show different pathology levels. In the SNc, the 3D registration led to SNc pathology being mis-assigned to other midbrain regions. In the PRC, no pathology is present, but a ventricular stain artifact leads to aberrant pathology calling in the 3D map. Scale bars = 250 μm. (I) Correlation plot highlighting thalamic regions. While pathology was generally well-represented in both 2D and 3D analysis, we identified PVT and XI thalamus regions that were not well-sampled by our 2D sampling strategy but were picked up by the 3D map. (J) The pathology in these regions (PVT, XI) was apparent with additional 2D sampling. Scale bars = 250 μm.

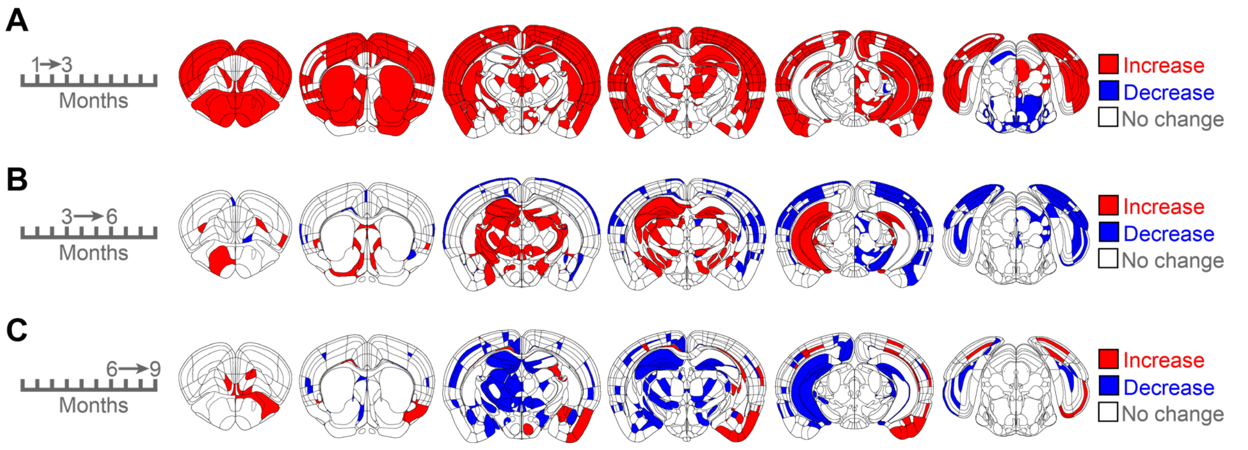

Figure S3. Time-dependent changes in regional α-synuclein pathology (A) Statistical comparisons were made comparing α-synuclein pathology in each region from 1 MPI to 3 MPI. Red indicates a statistically-significant increase in pathology (second generation *p* values), while blue indicates a statistically-significant decrease. Most regions have increased pathology at 3 MPI compared to 1 MPI. (B) Comparisons of α-synuclein pathology changes from 3 to 6 MPI. There are many fewer changes, with increases in contralateral hippocampus, but decreases in caudal cortical regions. (C) Comparisons of α-synuclein pathology from 6 to 9 MPI. Almost all regions have peaked and are stable or decreasing, with the exception of some amygdala regions and piriform, which are still increased.

Figure S4

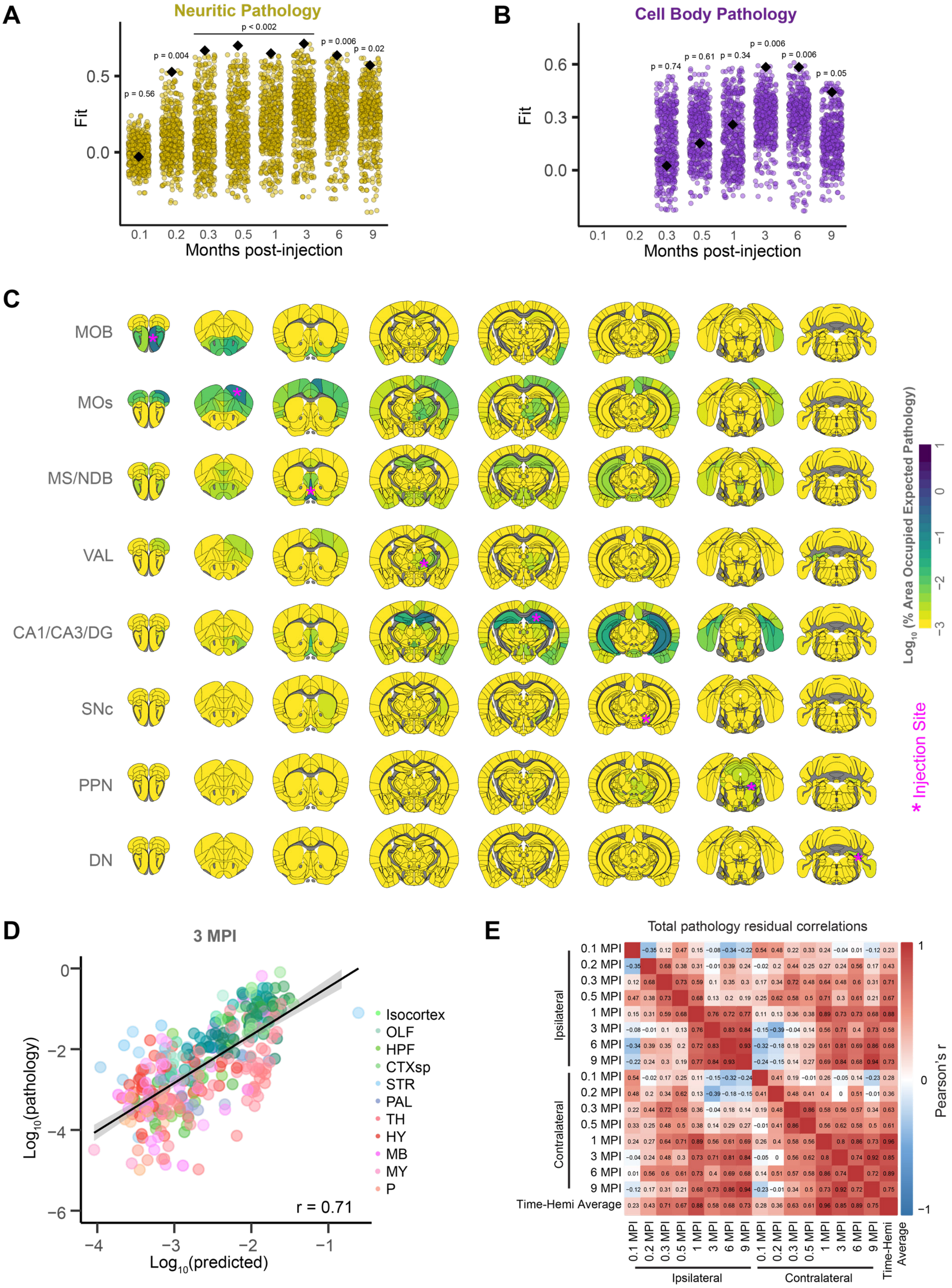

A
Neuritic Pathology
Fit
Months post-injection
p = 0.56
p = 0.004
p < 0.002
p = 0.006
p = 0.02
B
Cell Body Pathology
Fit
Months post-injection
p = 0.74
p = 0.61
p = 0.34
p = 0.006
p = 0.006
p = 0.05
C
MOB
MOs
MS/NDB
VAL
CA1/CA3/DG
SNc
PPN
DN
Log10 (% Area Occupied Expected Pathology)
* Injection Site
D
3 MPI
Log10(pathology)
Log10(predicted)
r = 0.71
Isocortex
OLF
HPF
CTXsp
STR
PAL
TH
HY
MB
MY
P
E
Total pathology residual correlations
Ipsilateral
Contralateral
Time-Hemi Average
Pearson's r

Figure S4. Computational model predictions of regional vulnerability Comparison of Pearson's *r* values obtained by fitting bidirectional spread models using actual (black diamond) and alternate (circles) seed regions for neuritic (A) or cell body (B) pathology. (C) Pathology predicted given linear diffusion modeling with the same parameters fit from the caudoputamen injection site, but with alternate injection site. MOB: main olfactory bulb, MOs: secondary motor area, MS/NDB: medial septal nucleus/diagonal band nucleus, VAL: ventral anterior-lateral complex of the thalamus, CA1/CA3/DG: hippocampus (CA1, CA3, dentate gyrus), SNc: substantia nigra-compact part, PPN: pedunculopontine nucleus, DN: dentate nucleus. (D) Model prediction of total pathology from Fig. 4A, but here plotted colored by major anatomical division. This demonstrates that certain anatomical divisions (thalamus, midbrain) have lower pathology than expected based on connectivity. (E) Heatmap displaying the linear regression correlations between residuals for brain regions derived from computational modeling of α-synuclein pathology at different timepoints and hemispheres. Residuals from 1-9 MPI show similar correlations and are hemispheres are also correlated at these timepoints.

Figure S5

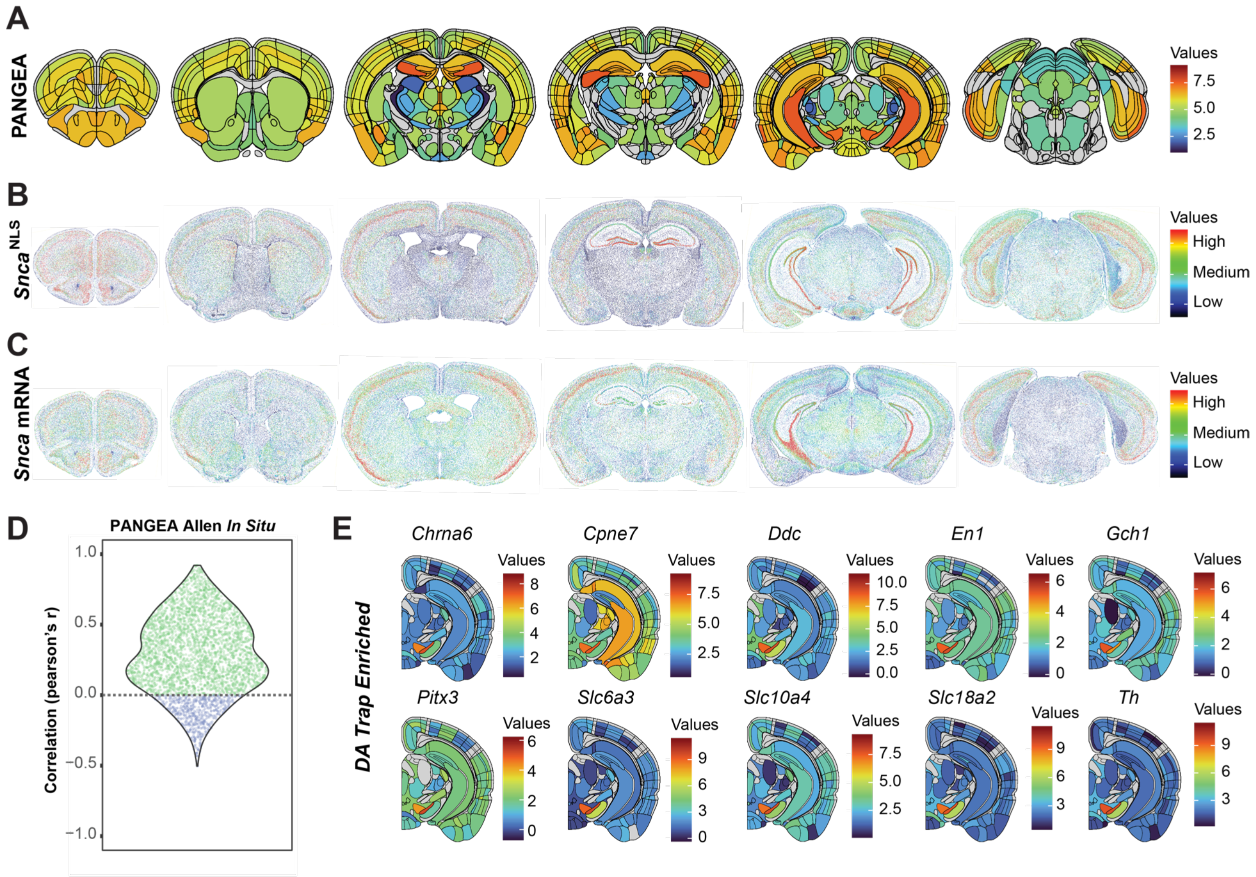

Figure S5. PANGEA comparisons to other gene expression atlases (A) Anatomical heatmap of *Snca* expression values in PANGEA compared to (B) protein staining of α-synuclein in mice where α-synuclein is tagged with a nuclear localization signal and (C) *Snca in situ* hybridization staining. Panels B and C display the relative expression of α-synuclein or *Snca* in each cell with warm values corresponding to high expression. Data adapted from PMID: 38504090. (D) Correlation of each gene contained in both Allen *in situ* atlas and PANGEA (Pearson's r). (E) PANGEA anatomical heatmaps of 10 genes previously identified as dopaminergic neuron selective using dopamine-driven RiboTRAP38 or enriched in the substantia nigra over the ventral tegmental area.

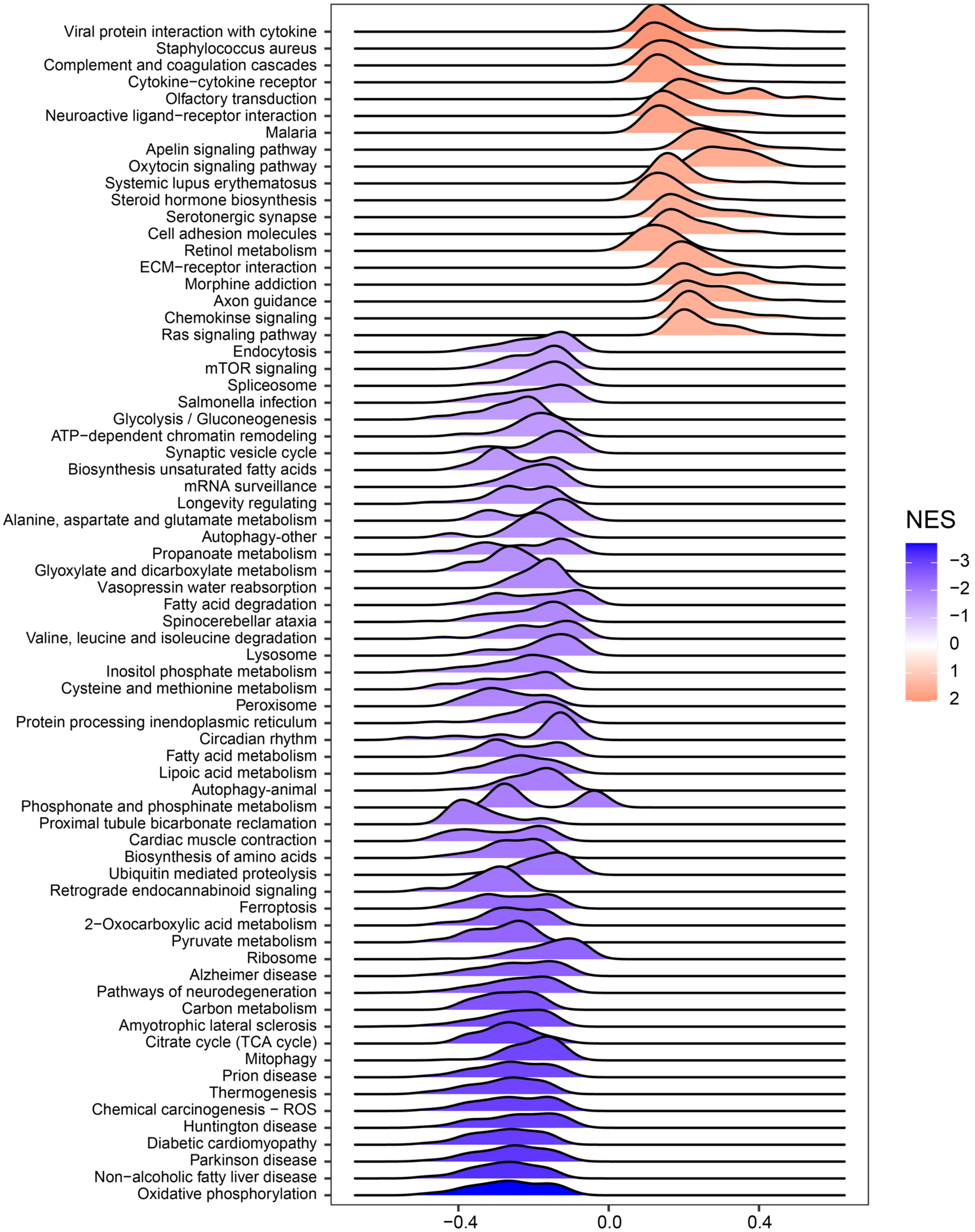

Figure S6. Gene set enrichment analysis significantly related to regional vulnerability

Ridgeplots of Normalized Enrichment Scores from gene set enrichment analysis of genes significantly correlated to pathology vulnerability (FDR<0.05)

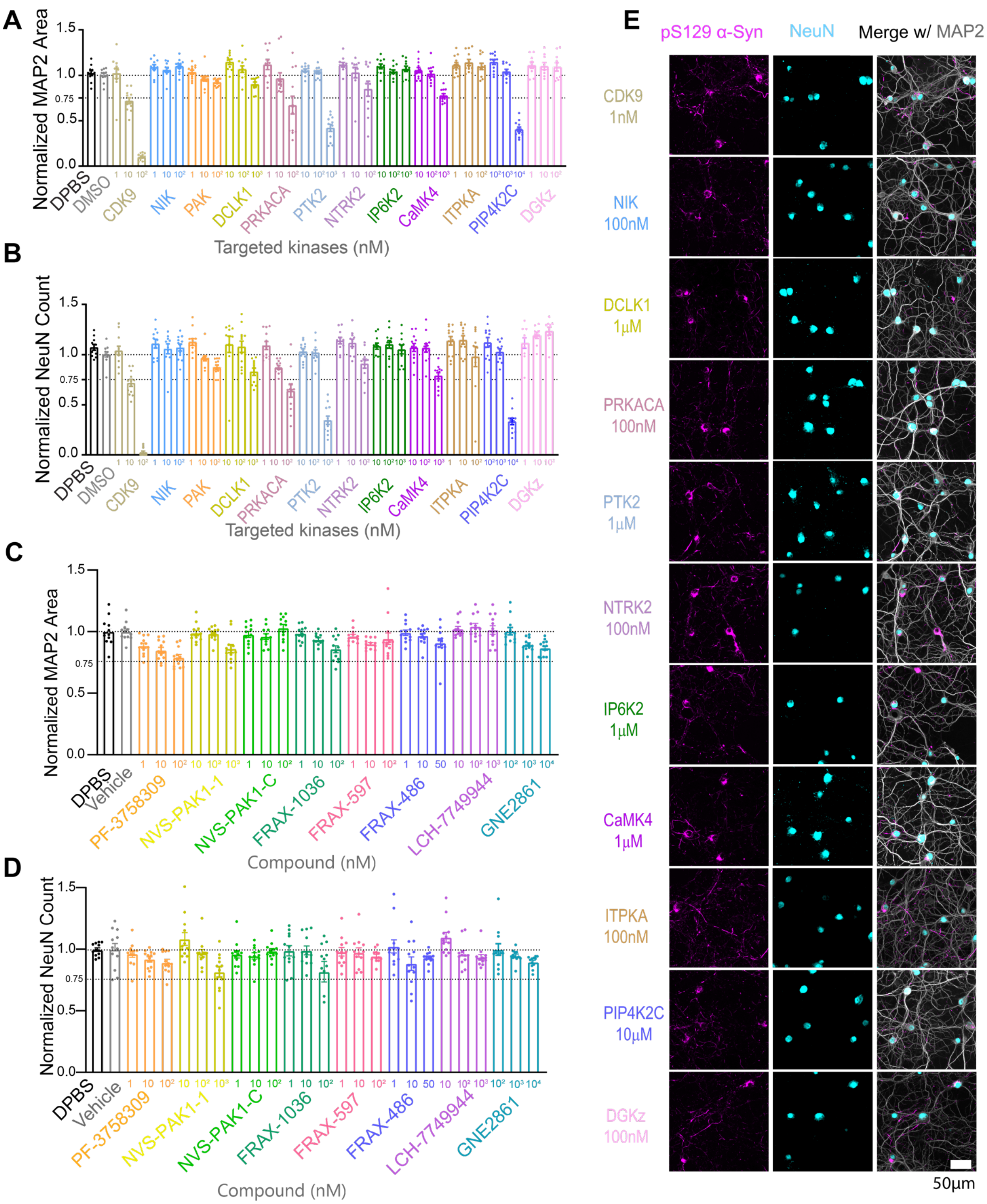

Figure S7. Effect of kinase inhibitors on neuronal health (A) MAP2 area and (B) NeuN count normalized to vehicle treated group from the kinases inhibited at 3 different doses in primary hippocampal neurons treated with α-synuclein PFFs from the screen of 12 kinase inhibitors. (C) MAP2 area and (D) NeuN count normalized to vehicle treated group from screen of various PAK inhibitors with varying specificity. Data presented as mean ± SEM with individual values plotted. N=12 independent wells from 4 separate cultures. (E) Representative images of

pS129 α-synuclein from the kinase inhibitor screen at the highest safe test dose for each kinase inhibitor. Scale bars = 50 μm.

Figure S8

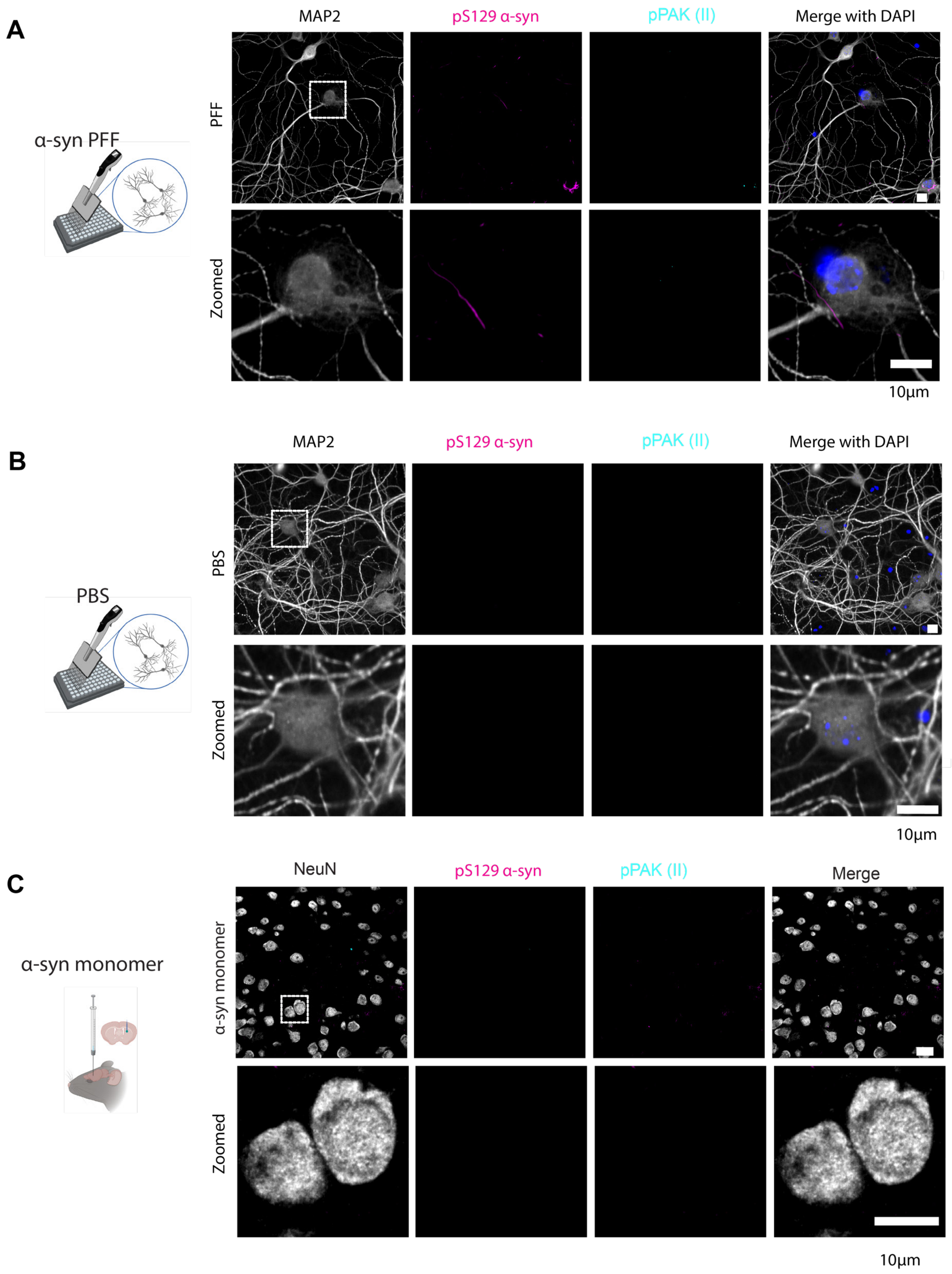

Figure S8. Group II pPAK in neurons without α-synuclein inclusions

(A) Representative images of co-immunofluorescence from primary neurons treated with α-synuclein PFFs with a zoom of a neuron without an α-synuclein inclusion. Note, this is the same field of view as Fig. 8H with a zoomed image of a different neuron. (B) Representative images of co-immunofluorescence from primary neurons treated with PBS showing no pPAK (group II) punctate pattern in cells without α-synuclein inclusions. (C) Representative images for co-immunofluorescence of α-synuclein inclusions and pPAK (group II) in amygdala from mouse injected with α-Syn monomer in dorsal striatum and stained 3MPI. Scale bars = 10 µm
